# Hierarchical Multi‐Dimensional Maturation Modeling to Isolate the Effects of Commercial Closure on a Great Lakes Fishery

**DOI:** 10.1111/eva.70075

**Published:** 2025-03-04

**Authors:** Zachary S. Feiner, Jason C. Doll, Ben D. Dickinson, Mark R. Christie

**Affiliations:** ^1^ Office of Applied Science, Wisconsin Department of Natural Resources Science Operations Center Madison Wisconsin USA; ^2^ Center for Limnology University of Wisconsin‐Madison Madison Wisconsin USA; ^3^ Freshwater Ecology Center, Department of Biology Francis Marion University Florence South Carolina USA; ^4^ Indiana Department of Natural Resources Michigan City Indiana USA; ^5^ Department of Biological Sciences Purdue University West Lafayette Indiana USA; ^6^ Department of Forestry and Natural Resources Purdue University West Lafayette Indiana USA

**Keywords:** commercial harvest, eco‐evo, fisheries‐induced evolution, freshwater fish, maturation

## Abstract

As anthropogenic disturbances rapidly change natural environments, species must respond to new selective pressures shaping rates of reproduction, growth, and mortality. One example is intense fisheries harvest, which can drive the evolution of heavily fished populations toward maturation at smaller sizes and younger ages. Changes in maturation have often been measured using probabilistic maturation reaction norms (PMRNs), which were originally designed to control for phenotypic plasticity while allowing for the detection of the evolution of maturation. However, multiple studies have highlighted issues with PMRN estimation, particularly with respect to their accuracy when parameterized with sparse data or when applied to populations experiencing myriad environmental stressors. We used a three‐decade time series of Laurentian Great Lakes yellow perch ( *Perca flavescens* Mitchill) data to develop a novel, hierarchical Bayesian PMRN estimation method that can explicitly account for these conceptual issues. Our results indicate that commercial fishing was a primary driver of maturation change in this population, and that the relaxation of harvest pressure via the closure of the commercial fishery in the late 1990s resulted in adaptation toward older ages and larger sizes at maturation within 2–3 generations. Future pairing of hierarchical Bayesian PMRN methods with genome‐wide data will help reveal the genetic underpinnings of maturation, and could lead to new avenues for integrating PMRNs into fisheries management and policy.

## 1 Introduction

As anthropogenic forces rapidly change natural environments, species are constantly responding to new pressures that shape rates of reproduction, growth, and mortality (Heath et al. 2003; Audzijonyte et al. 2016; Snell‐Rood and Ehlman 2021). Tradeoffs between these vital rates are critical to determining a range of individual and population‐level dynamics, for example, how early in life an individual begins to reproduce or the rate at which overexploited populations can replenish themselves (Lahti et al. 2009; Dunlop, Eikeset, and Stenseth 2015). Life history responses to stressors can often arise through phenotypic plasticity—however, a growing body of evidence has shown that rapid evolutionary processes can also shape the life history patterns of populations, including plasticity itself (Carroll et al. 2007; Crispo et al. 2010). One hallmark example of this rapid evolution is the intense commercial fishery for Atlantic cod ( *Gadus morhua* L.), where a fishery collapse coincided with rapid evolution toward maturation at small sizes and young ages with relatively limited recovery (Olsen et al. 2004; Barot et al. 2004b; Swain, Sinclair, and Hanson 2007). These evolutionary changes can have lasting effects on population viability, potentially resulting in a less productive fishery in addition to modifying the ability to respond to future stresses (Kuparinen and Hutchings 2012; Dunlop, Eikeset, and Stenseth 2015; Frank et al. 2016). Abrupt evolutionary shifts, therefore, can lead to novel population dynamics with altered sets of phenotypic traits (Walsh et al. 2006; Brady et al. 2019).

Harvest by humans, both recreationally and commercially, has consistently been posited to represent a significant selection pressure driving the evolution of myriad traits. Increased mortality rates brought on by intense harvest can themselves drive adaptation toward faster life histories, including earlier maturation (Heino, Díaz Pauli, and Dieckmann 2015). In addition, harvest by humans is also commonly size selective, which can impose strong selective gradients on harvested populations (Hendry, Farrugia, and Kinnison 2008). However, debate continues as to whether fisheries‐induced evolution represents a significant threat to harvested populations (Kuparinen and Festa‐Bianchet 2017; Kuparinen and Hutchings 2019). One possible reason for this equivocation is that the effects of harvest can act both through plastic and genetic mechanisms. Harvest can reduce population size, thereby increasing the body condition, growth, or reproductive success of surviving individuals by lessening intraspecific competition (Trippel 1995; Sass et al. 2004; Hutchings 2005). Size‐selective harvest, alternatively, can select against genotypes for fast growth or late maturity, either counteracting or enhancing, plastic changes in life history (Dieckmann and Heino 2007; Hendry, Farrugia, and Kinnison 2008). In addition, harvest is rarely the sole source of life history change. Temperature and food availability, acting via direct and indirect influences on growth and body condition, have been shown to exert strong influences on the age and size at which individuals become mature (Uusi‐Heikkilä et al. 2011; Feiner, Shaw, and Sass 2019). These forces can rapidly complicate the estimation and characterization of population life history traits, extending to our ability to predict the speed at which populations may respond to environmental or anthropogenic change. If responses are largely plastic, populations should be able to respond to, or recover from, stressors relatively rapidly. Conversely, responses with a strong genetic basis may take many generations for recovery, requiring both sufficient remaining genetic variation and countervailing selection gradients (Lahti et al. 2009; Allendorf, Berry, and Ryman 2014; Allen et al. 2018). Examinations of these processes have focused mostly on marine systems (e.g., Olsen et al. 2004; Swain, Sinclair, and Hanson 2007) or via experimental simulation of harvest (e.g., Atlantic silverside *Menidia menidia* L.; Conover and Munch 2002; Walsh et al. 2006; Conover, Munch, and Arnott 2009), with the notable exception of a series of studies in Windermere pike ( *Esox lucius* L.) showing the importance of the relative strengths of natural and anthropogenic selection in shaping life history evolution in a harvested population (Edeline et al. 2007, 2009).

Over the past two decades, probabilistic maturation reaction norms (PMRNs) have been developed to aid in distinguishing eco‐evolutionary patterns in individual maturation timing (Dieckmann and Heino 2007). Briefly, the concept rests on explicitly modeling the effects of growth, body size, and age on the maturation process, thereby accounting for plasticity in maturation arising from variation in growth. Variation in growth is assumed to be largely related to environmental conditions, such as food availability (Stearns and Koella 1986; Heino, Dieckmann, and Godø 2002). The residual variation in the size and age at which fish mature over time or among populations after accounting for plastic effects is then often assumed to at least partially represent adaptive or evolutionary variation in maturation schedules (Barot et al. 2004a; Dieckmann and Heino 2007). This assumption has been partially supported in an examination of changing allele frequencies in a historical analysis of Atlantic salmon ( *Salmo salar* L.) maturation schedules (Therkildsen et al. 2013). The PMRN approach has been used to suggest potential fisheries‐induced evolution in numerous fish stocks (e.g., Sharpe and Hendry 2009; Feiner et al. 2015b), and has been expanded to include plasticity from the effects of factors beyond size and age, including body condition and temperature (Grift et al. 2007; Uusi‐Heikkilä et al. 2011).

Despite their realized and potential utilities, PMRNs have some drawbacks that have limited their wide application. First, estimating PMRNs with sufficient certainty to observe temporal trends usually requires large datasets with hundreds of observations of immature and mature individuals, limiting their use in most freshwater fisheries that lack such high‐resolution data (Barot et al. 2004a). Second, as noted above, the myriad possible environmental factors that influence maturation means interpreting changes in PMRNs as evolutionary can often be tenuous, especially in highly dynamic environments. Here, we leverage a 30‐year time series of yellow perch ( *Perca flavescens* Mitchill) data from Lake Michigan, USA, to develop a hierarchical Bayesian method for modeling PMRNs, with potentially widespread applications for modeling PMRNs throughout the region. Lake Michigan yellow perch once supported a high‐value commercial fishery in the Great Lakes before intense fishing pressure precipitated a rapid collapse of the fishery in the early to mid‐1990s. In response, commercial fishing was closed in 1997 and recreational fisheries were highly restricted (Wilberg et al. 2005). Despite these actions, yellow perch has shown little recovery in Lake Michigan (Redman et al. 2011). Previous work revealed rapid, large changes in PMRNs toward maturation at larger sizes and older ages in Lake Michigan yellow perch following the closure of commercial fishing—however, that work was limited in temporal resolution (PMRNs were measured on the decadal scale) and did not account for multiple environmental changes occurring at the same time as changes in yellow perch populations (Feiner et al. 2015b, 2017). For example, changes in water quality regulation, followed by the invasion of dreissenid mussels, significantly decreased system productivity in the 2000s, while climate change has warmed surface waters (Bunnell et al. 2014; Collingsworth et al. 2017). It thus remains unknown to what extent the rapid changes in maturation previously observed in this stock were due to plastic or evolutionary processes, and what role these changes may play in the inability of the stock to recover post‐collapse. Therefore, Lake Michigan yellow perch provides an exemplary system in which improved, multivariate PMRN estimation could yield a deeper understanding of the mechanisms for fishery collapse and post‐collapse dynamics.

## 2 Methods

### 2.1 Biotic Data

**FIGURE 1:**
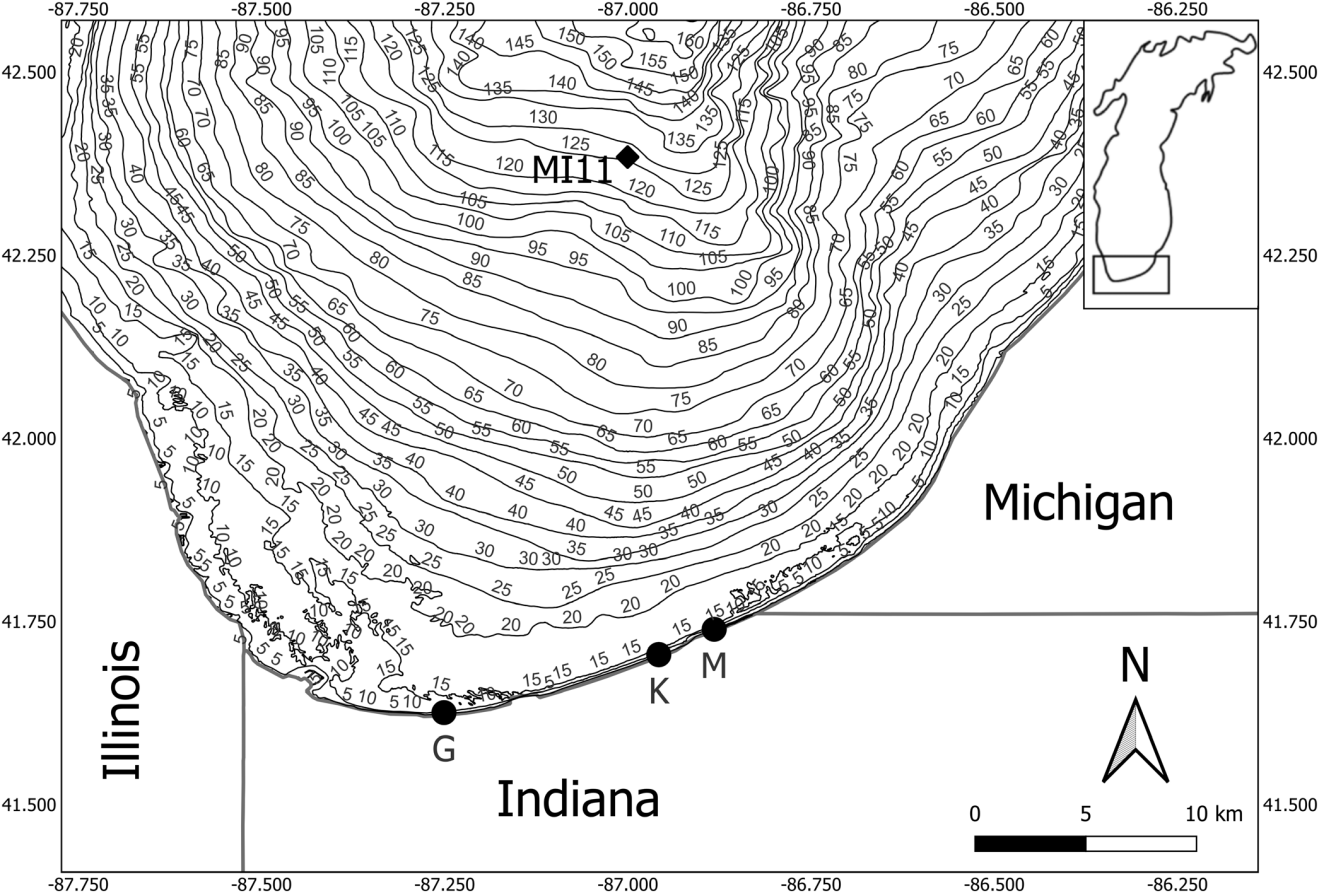
Map of southern Lake Michigan, USA, showing depth contours (m) and fish sample sites for yellow perch from 1984 to 1988 (sites K and M) and 1989 to 2016 (sites G, K, and M) and water quality sample site (MI11).

**FIGURE 2:**
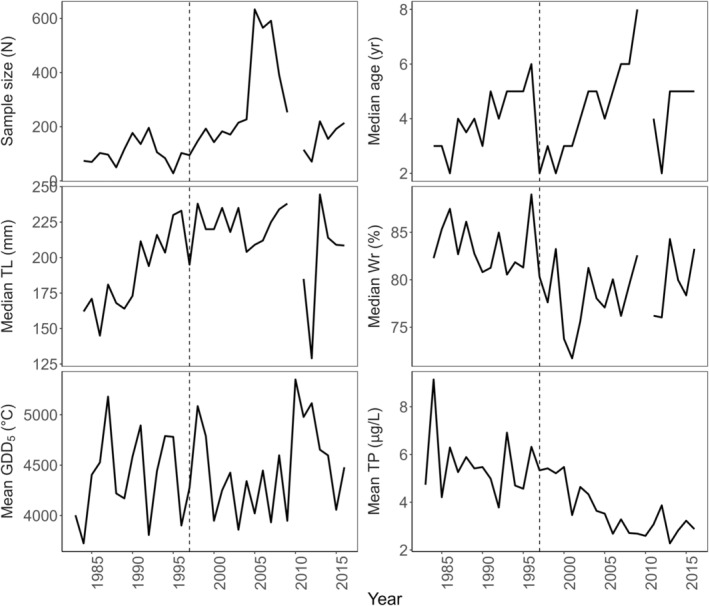
Time series of explanatory variables used to model southern Lake Michigan female yellow perch maturation, including per year sample size, age, total length (mm TL), relative weight (Wr), growing degree days (GDD_5_), and total phosphorus (TP). Dashed line denotes the closure of the commercial fishery.

The yellow perch population in this study represents a single, large, panmictic population residing in southern Lake Michigan (Schraidt et al. 2023). Yellow perch were collected using nighttime bottom trawls along the 5‐m depth contour at two (1984–1988, sites K and M) or three (1989–2016, sites K, M, and G) sites semi‐monthly from June to August with a standard semi‐balloon bottom trawl with 4.9 m headrope, 5.8 m footrope, 38.1 mm stretch mesh body, and 12.7 mm stretch mesh cod end (Figure 1). Total effort varied over time in response to the effort needed to assess abundance as yellow perch populations declined, ranging from 12 to 34 h of trawling across sites (Shroyer and McComish 1998; Lauer et al. 2008). Experimental gillnets were used in conjunction with trawling. Between 1984 and 2010 experimental gillnets consisted of nine repeating panels of 51‐, 64‐, and 76‐mm stretch mesh ~15 m each in length. Additional panels consisting of 25‐, 32‐, 38‐, and 44‐mm stretch mesh were added in 2011 for a total of 21 panels each ~15 m in length. Each set was deployed at approximately 1900 h and pulled at approximately 0700 h the next day. Total annual effort ranged from 8 to 36 net nights. Up to 300 fish were randomly selected and measured for total length (TL, mm) and weighed (g) during each sampling event. Sex and maturation status were determined via internal assessment of gonads. Up to 10 fish per 10 mm length class were randomly selected and aged using either scales (primarily before 1994) or opercular bones (primarily after 1994; Baker and McComish 1998). Scales were dried and stored in coin envelopes before and after aging, whereas opercles were boiled before storage. Structures were aged by two readers and any discrepancies were reviewed before a consensus age was reached. The change in aging protocol was implemented following a study that showed a slightly lower coefficient of variation in ages assigned from opercles than scales (Baker and McComish 1998). While neither method has been validated for yellow perch, Robillard and Marsden (1996) showed that underestimation of ages from scales compared to otoliths began at ages older (> 7 years) than the focal ages in this study. Scales or opercles were removed from 15,975 male and 30,520 female yellow perch between 1984 and 2016, averaging 1367 per year (range 224–2922). Relative weights (Wr) were calculated as Wr = *W*/Ws * 100, where *W* is the observed weight of the fish and Ws is the standard weight. Standard weights were calculated as log_10_(Ws) = −5.386 + 3.230log_10_(TL), based on the equation derived from the length–weight relationship for yellow perch from 78 populations developed by Willis, Guy, and Murphy (1991). Weights for unweighed fish were predicted from year‐specific length‐weight relationships based on data for all fish that were measured and weighed. All yellow perch data are published and publicly available in the Purdue University Research Repository (Feiner et al. 2015a; Figure 2).

### 2.2 Abiotic Data

We chose to include indices of temperature, productivity, and fishing in the model of yellow perch maturity to account for their potential effects on the maturation process (Figure 2). Temperature, via influences on food availability and metabolic or growth rates, may be predicted to increase the probability of maturation (Tobin and Wright 2011; Kuparinen et al. 2011). Similarly, additional food availability brought about by increasing primary productivity could influence growth rates, body size, or excess energy reserves to increase the probability of maturation (Henderson and Morgan 2002; Feiner, Shaw, and Sass 2019). Temperature and total phosphorus ranged considerably over time (Figure 2), meaning accounting for their potential effects on maturation was important to better disentangle the effects of environmental conditions on maturity status.

Commercial fishing for yellow perch was closed in 1997 and has been hypothesized to have contributed to rapid changes in yellow perch maturation schedules in the subsequent years (Feiner et al. 2015b). However, we lacked estimates of selection gradients, fishing mortality, or exploitation rates on annual timesteps to include in the model. Therefore, we simply indexed fishing pressure as a binary categorical variable (during commercial fishing = 1, all years before 1997, after the closure of commercial fishing = 0, all years including and after 1997). Annual growing degree days (base temperature 5°C, GDD) were calculated from daily water temperatures provided by the St. Joseph Water Filtration Plant in St. Joseph, Michigan (Forsythe, Doll, and Lauer 2012; Water Plant Superintendent, http://www.sjcity.com; Figure 2). Total phosphorus (TP, ug/L) data were obtained from the EPA Great Lakes National Program Office Great Lakes Environmental Database (GLENDA) from site MI11 located in southern Lake Michigan. Total phosphorus data were collected in spring and summer each year, and we averaged all yearly observations to generate a time series of mean total phosphorus to index system productivity (Figure 2).

### 2.3 Modeling Growth and Maturity

Size and age at maturation is a highly plastic trait in many fish species that responds to variance in individual growth and mortality rates, body condition, temperature, and other ecological factors (Dieckmann and Heino 2007). Most PMRNs focus on age and size (length) in a two‐dimensional framework (e.g., Feiner et al. 2015b, 2017); however, previous work suggests that body condition may also be a relevant factor influencing individual maturation in percids (Henderson and Morgan 2002; Feiner, Shaw, and Sass 2019) and other fishes (Grift et al. 2007; Mollet, Kraak, and Rijnsdorp 2007). Therefore, we used age, length, and body condition (indexed as relative weight, Willis, Guy, and Murphy 1991) to model maturation as a multi‐dimensional PMRN to better account for ecological plasticity in maturation. Moreover, the effects of age, length, and condition on maturation are likely not independent due to fitness tradeoffs and correlations among traits as fish grow and age (Marty et al. 2011; Christie et al. 2018; Feiner, Shaw, and Sass 2019), meaning accounting for interactions among the effects of these variables is critical for appropriately capturing their effects on maturation.

We used a hierarchical Bayesian approach to test the effects of age, length, condition (relative weight), water temperature, total phosphorus, and commercial fishing on the maturity status of female yellow perch. We focused on females because of previous research documenting significant shifts in female PMRNs (Feiner et al. 2015b), as well as the importance of female life history traits to fish population productivity (Hixon, Johnson, and Sogard 2013; Feiner, Shaw, and Sass 2019). In addition, male fish in this population are often mature before they become susceptible to sampling gear, leading to catches that are mostly or entirely mature and poor performance of models for male maturation (Feiner et al. 2015b).

A benefit of the Bayesian approach is that uncertainty in estimates can be directly derived from posterior distributions of parameter estimates, allowing for the propagation of uncertainty in the maturity and growth models throughout the modeling process (Wright, Millar, and Gibb 2011; Feiner et al. 2015b). While Bayesian methods have previously been used to fit PMRN models (Wright, Millar, and Gibb 2011; Feiner et al. 2015b, 2017; Renneville et al. 2020), to the best of our knowledge, a full examination of hierarchical Bayesian models has not been applied to PMRN estimation. Therefore, our development of this method could be widely applicable in fisheries where maturity data and sample size of some groups (e.g., cohorts) do not meet the thresholds required for previously described frequentist or bootstrapping methods.

The model estimates cohort‐ and age‐specific PMRNs using a hierarchical model in which we first determine the probability of maturity at a given set of covariates, using multiple logistic regression including interactions among age, length, and condition:

$$\begin{array}{c} \text{logit}\;\left(o_{c,i,a,l,w,f,t,p}\right)=\beta_{1\left[c\right]}+\beta_{2\left[c\right]}a_{i}+\beta_{3\left[c\right]}l_{i}+\beta_{4\left[c\right]}w_{i}\\ +\beta_{5\left[c\right]}a_{i}*l_{i}+\beta_{6\left[c\right]}a_{i}*w_{i}+\beta_{7}f_{i}+\beta_{8\left[c\right]}t_{i}+\beta_{9\left[c\right]}p_{i} \end{array}$$

$$y_{c,i,a,l,w,f,t,p}\sim \text{Bernoulli}\;\left(o_{c,i,a,l,w,f,t,p}\right)$$

where $o_{c,i,a,l,w,f,t,p}$ is the probability of individual *i* from cohort *c* being mature given covariates; $\beta_{1:9\left[c\right]}$ are the coefficients of fixed effects and interactions for cohort *c*, including (1) model intercept; (2) $a_{i}$ is the age (years) of individual *i*; (3) $l_{i}$ is the total length (mm) of individual *i*; (4) $w_{i}$ is the relative weight of individual *i*; (5) the interaction between total length and age; (6) the interaction between relative weight and age; (7) $f_{i}$ is a dichotomous variable for commercial fishing (1 = commercial fishing occurring from 1983 to 1996 and 0 = no commercial fishing occurring from 1997 to 2015); (8) $t_{i}$ is the growing degree days; and (9) *p*_*i*_ is total phosphorus. The response $y_{i}$ is a dichotomous variable representing mature (1) or immature (0). With the exception of the effect of fishing (due to its correlation with time as a binary variable), the effects of all covariates and interactions are continuous and nested within a hierarchical effect of cohort, as cohorts could experience variable thermal and productivity gradients during their lifespan (Figures S1 and S2). For example, the effect of age for cohort *i* was derived from a normal distribution $\beta_{2\left[c\right]}\sim N\left(\mu_{2},\sigma_{2}\right)$ with hyperpriors for the mean effect, $\mu_{2}$, and standard deviation of effects, $\sigma_{2}$ (Table 1). All continuous covariates were standardized to *z*‐scores prior to analysis.

**TABLE 1:** Prior probability distributions were used in the maturation model.

Parameter	Description	Prior probability distribution
$\beta_{1:9\left[c\right]}$	Coefficients 1 through 9 of fixed effects for cohort *c* in the maturation model	Normal $\left(\mu_{1:9},\sigma_{1:9}\right)$
$\mu_{1:9}$	Mean of nine fixed effects in the maturation model	Normal $\left(0,10\right)$
$\sigma_{1:9}$	Standard deviation of nine fixed effects in the maturation model	Cauchy $\left(0,5\right)$ ^+^
$\varphi_{\left[c\right]}$	Intercept for cohort *c* in the growth model	Normal $\left(\mu_{\varphi },\sigma_{\varphi }\right)$
$\mu_{\varphi }$	Mean of the cohort intercepts in the growth model	Normal $\left(100,20\right)$
$\sigma_{\varphi }$	Standard deviation of cohort intercepts in the growth model	Cauchy $\left(0,5\right)$ ^+^
$\gamma_{\left[c\right]}$	Coefficient for age effects for cohort *c* in the growth model	Normal $\left(\mu_{\gamma },\sigma_{\gamma }\right)$
$\mu_{\gamma }$	Mean of the age effects in the growth model	Normal $\left(100,20\right)$
$\sigma_{\gamma }$	Standard deviation of the age effects in the growth model	Cauchy $\left(0,5\right)$ ^+^
τ	Standard deviation of the growth model	Cauchy $\left(0,5\right)$ ^+^
*ω*_1:4[*c*]_	Coefficients 1 (intercept), 2 (age), 3 (length), and 4 (age‐length interaction) of fixed effects by cohort *c* in the body condition model	Normal (0,1)
*ζ*	Standard deviation of the body condition model	Cauchy $\left(0,5\right)$ ^+^

*Note:* “+” indicates the positive values of the Cauchy distribution.

Growth was modeled as a linear‐log regression, which provided a similar fit to length‐age data while being computationally simpler than other more complex growth models like the von Bertalanffy (Figure S3). This model was used to derive the total length at previous ages during the calculation of the PMRN (see below).

$$\mu_{i}=\varphi_{\left[c\right]}+\gamma_{\left[c\right]}\log\left(a_{i}\right)$$

$$l_{i}\sim \text{Normal}\;\left(\mu_{i},\tau \right)$$

where $\varphi_{\left[c\right]}$ and $\gamma_{\left[c\right]}$ are coefficients of the growth model for each cohort *c*; $\mu_{i}$ is the predicted length of individual *i* at age *a*. The effects of age and the intercept are continuous and nested within a hierarchical effect of cohort. For example, the effect of age for cohort *i* was derived from a normal distribution $\gamma_{\left[c\right]}\sim N\;\left(\mu_{\gamma },\sigma_{\gamma }\right)$ with hyperpriors for the mean effect $\mu_{\gamma }$ and standard deviation of effects $\sigma_{\gamma }$ (Table 1). Growth increments between ages were calculated using the growth model to predict the mean change in length (*∆l*) between age *a* and age *a* − 1 for each cohort.

Body condition likely varies over years owing to changes in food availability or other conditions influencing resource acquisition, such as body size or age. Therefore, we modeled mean body condition (denoted as *w*_*c,i,a,l*_) as a function of cohort, age, and length:

$$\theta_{c,i,a,l}=\omega_{1\left[c\right]}+\omega_{2\left[c\right]}a_{i}+\omega_{3\left[c\right]}l_{i}+\omega_{4\left[c\right]}a_{i}l_{i}$$

$$w_{c,i,a,l}\sim \text{Normal}\;\left(\theta_{c,i,a,l},\zeta \right)$$

where *ω*_1:4[*c*]_ are cohort‐specific coefficients for the intercept, and effects of age (*a*_*i*_), length (*l*_*i*_), and their interaction (*a*_*i*_ *l*_*i*_), *θ*_*c,i,a,l*_ is the predicted Wr of individual *i* from cohort *c* at its age *a* and length *l*, and ζ is a model error. Here cohort was treated as a fixed, rather than hierarchical, variable, while age and length were continuous covariates, such that the priors for *ω* were all derived from normal distributions *ω*_1:4[*c*]_ ~ *N*(0,1). Body condition for fish *i* from cohort *c* at previous ages (*a* − 1) and lengths (*l* − *∆l*) were then calculated as:

$$w_{c,i,a-1,l-∆l}=\omega_{1\left[c\right]}+\omega_{2\left[c\right]}\left(a_{i}-1\right)+\omega_{3\left[c\right]}\left(l_{i}-∆l\right)+\omega_{4\left[c\right]}\left(a_{i}-1\right)\left(l_{i}-∆l\right)$$

and the predicted incremental change in body condition from the previous year to the current year was calculated as *∆w* = $w_{c,i,a,l}$ − $w_{c,i,a-1,l-∆l}$.

The full posterior probability distribution was used to predict the probability that fish *i* was mature in its present state $\left(o_{c,i,a,l,w,f,t,p}\right)$ and the probability it was mature in its state the previous year $\left(o_{c,i,a-1,l-\mathrm{\Delta l},w-∆w,f-1,t-1,p-1}\right)$ based on estimations of changes in age, length, and body condition, and experience of variable fishing pressure, growing degree days, and total phosphorus by the cohort over time (Figures S1 and S2). Specifically, to account for their effects on the annual probability of a fish being mature via effects on growth or energetic state, for each fish we used the previous year's measures of age (*a* − 1), prediction of length at the previous age (*l* − *∆l*), prediction of body condition at the previous age and length (*w* − ∆*w*), and the previous year temperature (*t* − 1), fishing (*f* − 1), and phosphorus (*p* − 1).

### 2.4 Estimating Probabilistic Maturation Reaction Norms

The probability that fish *i* from cohort *c* first became mature at its current age, size, and condition, and given its experiences of temperature, fishing, and total phosphorus (*m*_*c,i,a,l,w,f,t,p*_) was calculated as:

$$m_{c,i,a,l,w,f,t,p}=\frac{\left(o_{c,i,a,l,w,f,t,p}-o_{c,i,a-1,l-\mathrm{\Delta l},w-∆w,f-1,t-1,p-1}\right)}{\left(1-o_{c,i,a-1,l-\mathrm{\Delta l},w-∆w,f-1,t-1,p-1}\right)}$$

PMRNs are often described and visualized using their midpoints in age‐length space (the age‐specific size at which a fish has a 50% probability of maturation, Lp_50_; Barot et al. 2004a). Midpoints were estimated using logistic regression (Barot et al. 2004b; Wright, Millar, and Gibb 2011; Feiner et al. 2015b). This included setting negative maturation probabilities to zero (i.e., if a fish had a higher probability of being mature the previous year than in the current year, $o_{c,i,a,l,w,f,t,p}-o_{c,i,a-1,l-\mathrm{\Delta l},w-∆w,f-1,t-1,p-1}$ < 0, and it was unlikely for it to be first maturing under current conditions). Logistic regressions using *m*_*c,i,a,l,w,f,t,p*_ as the response and *l*_*i*_ as the predictor were then fit within each cohort and age class on each draw of *m*_*c,i,a,l,w,f,t,p*_ from the model posterior. Regressions that would have included less than two fish or resulted in negative regression slopes (i.e., the probability of first maturation declining with length, meaning fish had likely already matured) were excluded. The midpoint (Lp_50_) was calculated as the ‐intercept/slope of the logistic regression, and the median and 95% credible intervals of midpoints were calculated from all posterior draws. Assuming reliable model fits, this method thus generated 6000 draws from the posterior distributions of Lp_50_ for each age and cohort from which to summarize PMRN shapes and shifts over time in age‐length space after accounting for the effects of interannual variation in condition, fishing, temperature, and productivity (similar to Uusi‐Heikkilä et al. 2011). Cohorts and ages where more than 5% of posterior draws were excluded based on the criteria above were excluded from further analysis to retain only estimates of midpoints that could be reliably estimated based on the data and model.

All analyses were performed in programs Stan (Carpenter et al. 2017) and R 4.2.3 (R Core Team 2022) via the RStan package (Stan Development Team 2022). Three concurrent Markov Chain Monte Carlo (MCMC) chains were used, each with 4000 iterations and discarding the first 2000 iterations. The final joint posterior probability distribution consisted of 6000 total iterations. Convergence was assessed using two methods. Split R‐hat compares variation of parameter estimates within chains to variation of the same parameter among chains, and with a convergence criterion of R‐hat < 1.1 (i.e., approximately equal variation within and among chains) for all parameters. Second, trace plots of all parameters were visually evaluated to examine mixing (Figures S4–S11). As a way to evaluate change in age‐specific Lp_50_ over time beyond the visual interpretation of the time‐series plots, the proportion of posterior overlap was calculated for cohorts near the beginning of the time series (pre‐1985), around the time the commercial fishery was closed (1995–1999), and at the end of the time series (2011–2014). Low posterior overlap would suggest that Lp_50_ differed between cohorts. The precise cohorts used varied among ages due to variability in the quality of model fitting.

## 3 Results

In total, the maturity dataset included observations from 6115 female yellow perch, of which 2571 were immature and 3544 were mature, covering 37 cohorts from 1979 to 2015 (Figure S12). Fish total lengths ranged from 46 to 385 mm (median = 208 mm) and ages ranged from 1 to 16 (median = 4). The number of fish sampled per year varied considerably over time—early years (from 1979 to the mid‐1990s) were generally represented by less than 200 fish per year, whereas in the early 2000s catches increased to ~600 fish per year before declining again to less than 200 fish per year (Figure 2).

**FIGURE 3:**
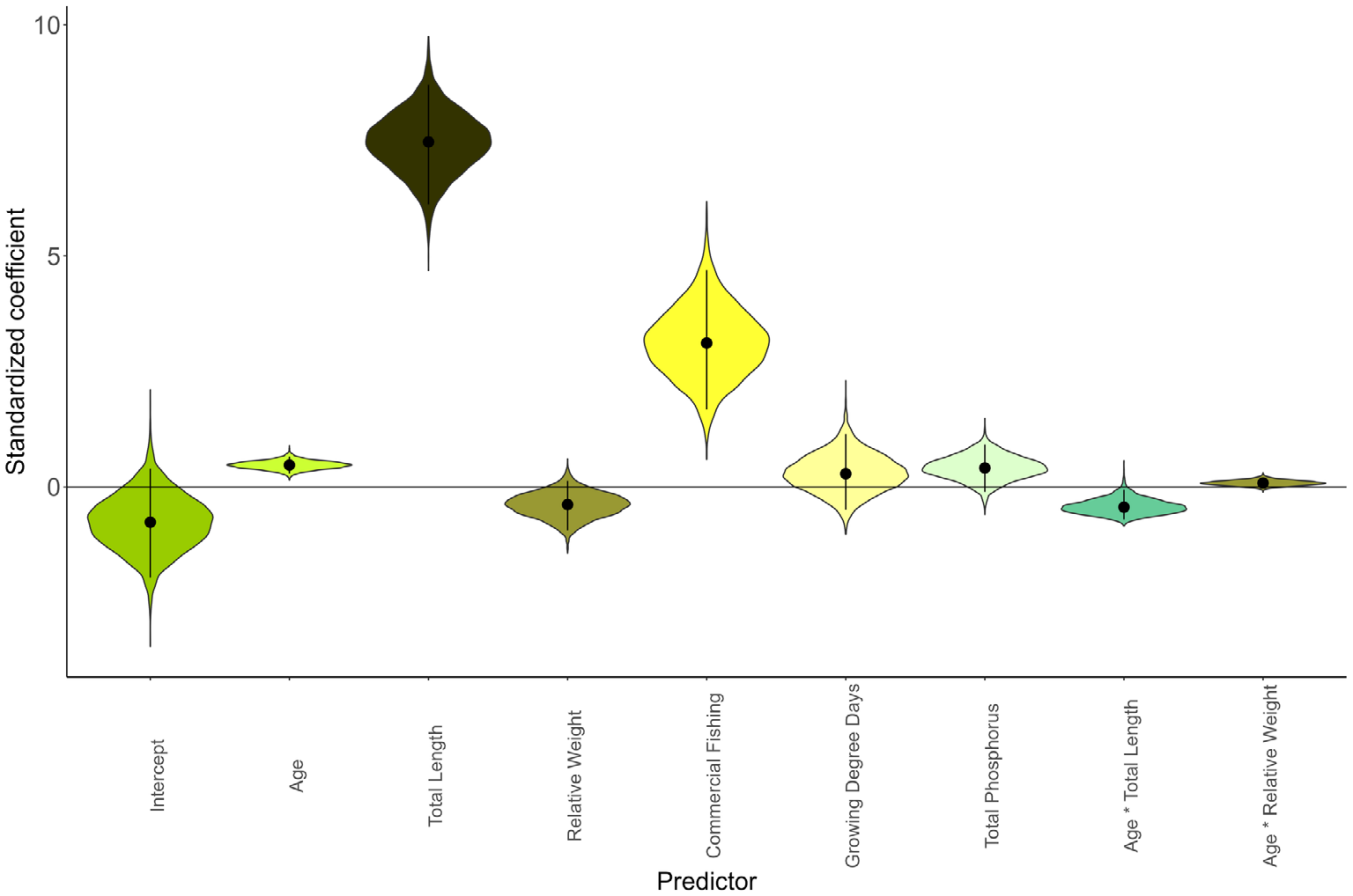
Posterior distributions of standardized model coefficients (variables scaled to mean = 0, SD = 1) for biotic and abiotic parameters included in hierarchical Bayesian estimation of southern Lake Michigan yellow perch probabilistic maturation reaction norms. Violin plots represent the distribution of 6000 draws from the joint posterior, point and whiskers are median and 95% credible interval, horizontal dotted line denotes zero.

Age, length, body condition, fishing, and growth environment (GDD and TP) had varying effects on the probability of maturity (Figure 3). Length had a clear, strong positive effect on maturity probability (*β* = 7.450, 95% credible interval [CI] = 6.118–8.706) whereas age had a small but clearly positive effect (*β* = 0.472, CI = 0.286–0.659), each of which was consistent across cohorts. Relative weight, an index of body condition, had negligible effects on maturity probability (*β* = −0.392, CI = −0.941 to 0.128). The effect of age was also weakly moderated by interaction with total length (*β* = −0.425, CI = −0.705 to −0.062), becoming slightly weaker at larger sizes, but there was not a clear interaction between age and body condition (*β* = 0.083, CI = −0.023 to 0.200).

**FIGURE 4:**
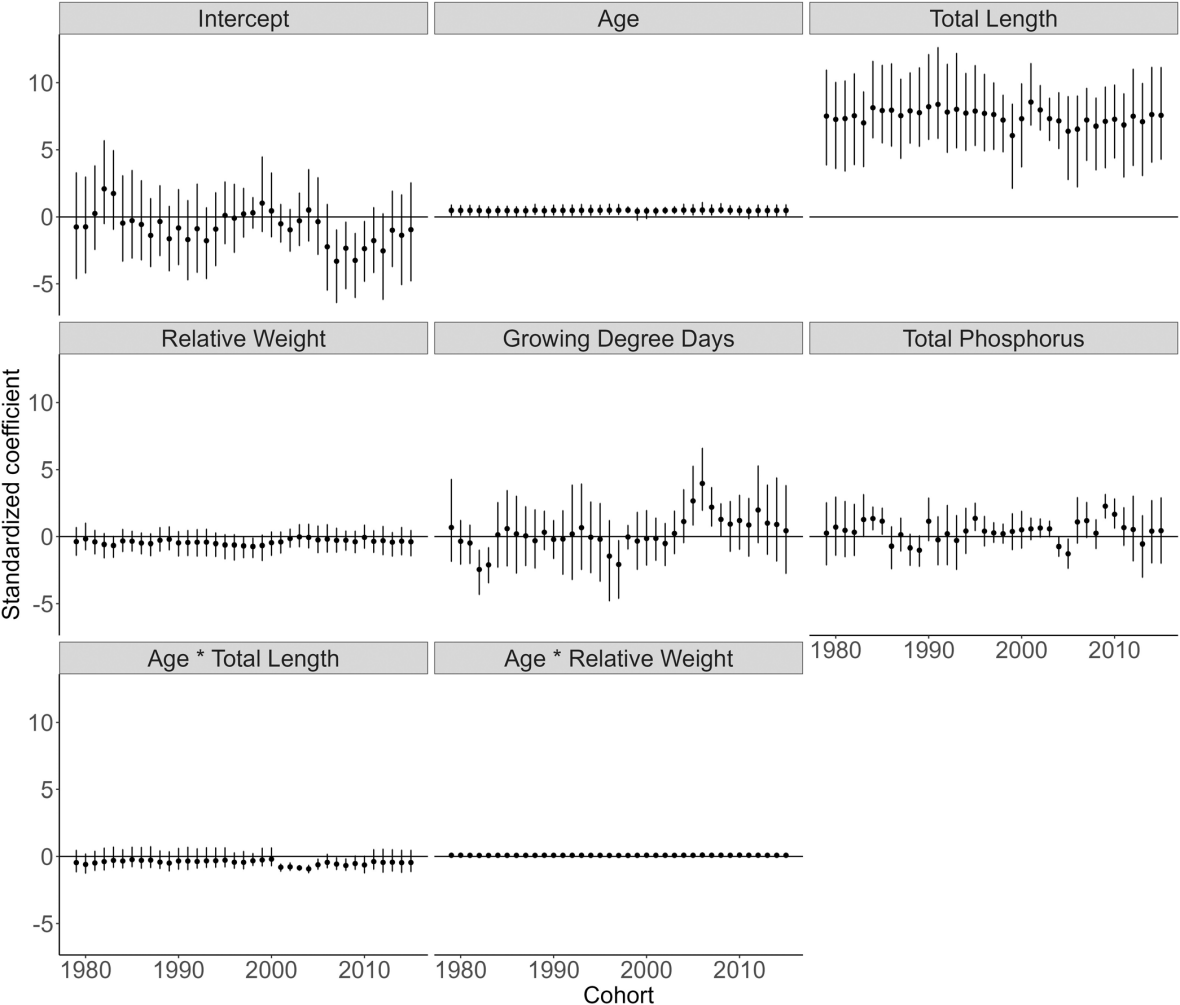
Cohort‐specific standardized (variables scaled to mean = 0, SD = 1) coefficient estimates (median and 95% credible intervals) for abiotic and biotic predictors of southern Lake Michigan yellow perch probabilistic maturation reaction norms from 6000 draws from the joint posterior of the hierarchical Bayesian model.

Among anthropogenic and environmental effects, the existence of commercial fishing was clearly positively related to maturity probability and had the second strongest effects of all predictors (*β* = 3.128, CI = 1.672–4.693; Figure 3). While the mean effect of GDD was negligible (*β* = 0.411, CI = −0.109 to 0.914), there was substantial variability in the importance of GDD among cohorts, where fish from years after ~2000 expressed a positive effect of GDD (Figure 4). Total phosphorus also had weak and variable effects on maturity status (*β* = 0.296, CI = −0.497 to 1.142; Figure 4).

**FIGURE 5:**
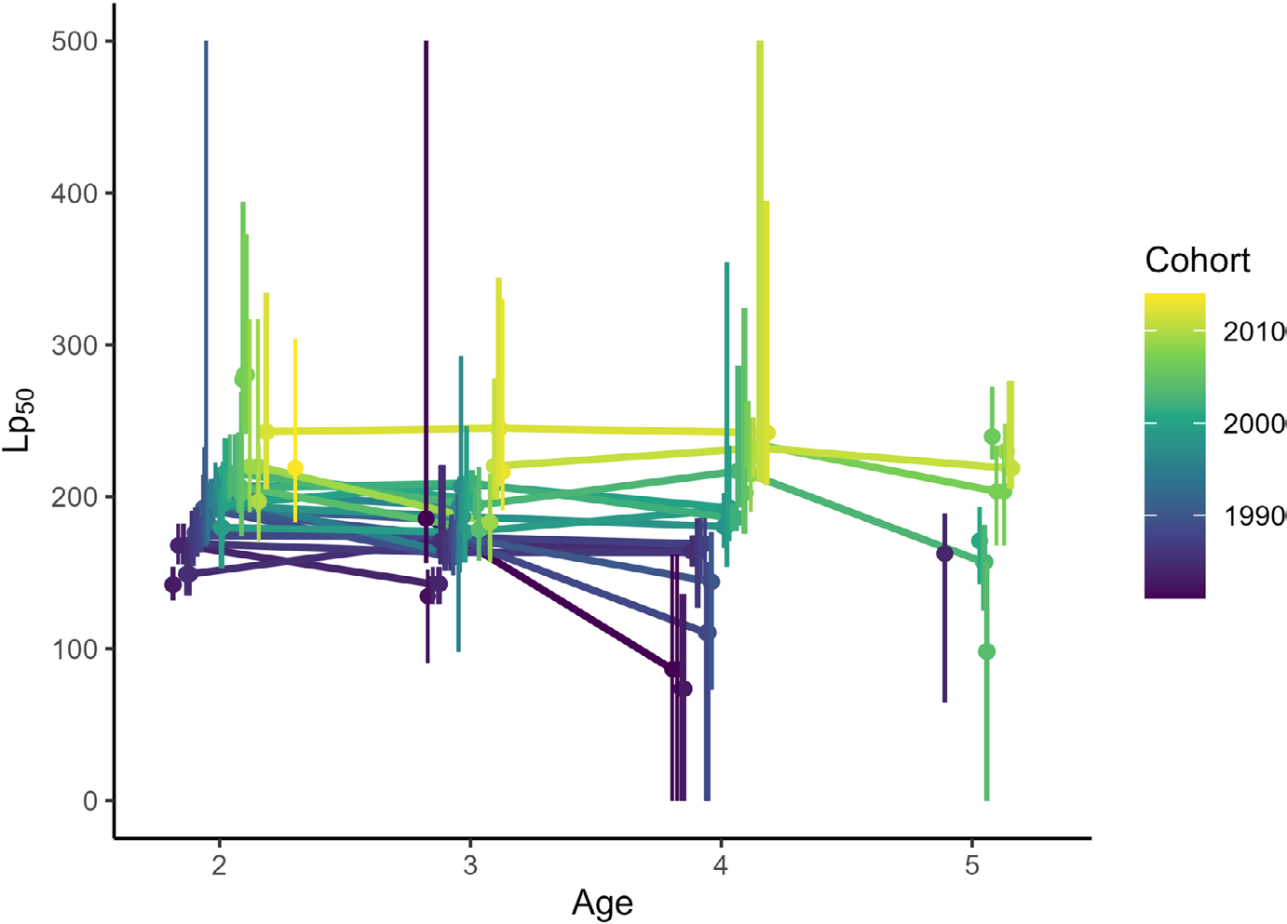
Estimates of probabilistic maturation reaction norm midpoints (Lp_50_) of southern Lake Michigan female yellow perch, color‐coded by cohort across ages, with more recent cohorts in brighter colors. Points represent medians, error bars are 95% credible intervals.

**FIGURE 6:**
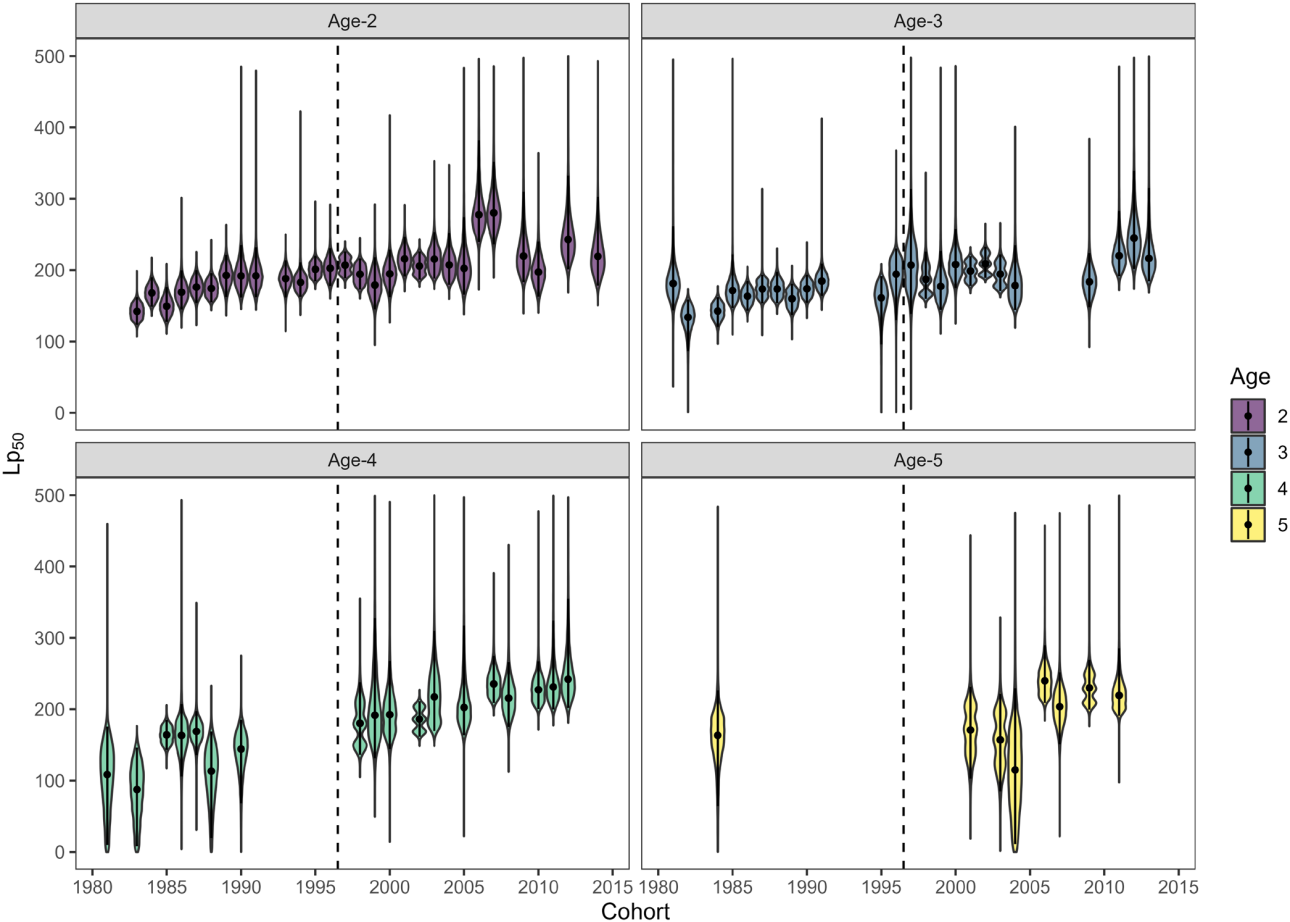
Posterior distributions of probabilistic maturation reaction norm midpoints (Lp_50_) of southern Lake Michigan female yellow perch by age (panels) to demonstrate temporal changes in the length at which ages reached 50% maturation probability. Points represent medians, vertical lines are 95% credible intervals, and the dashed vertical line denotes the year when the commercial fishery in Lake Michigan was closed.

After accounting for the effects of phenotypic, fishing, and environmental conditions on maturity, clear temporal trends remained in the PMRN midpoints for female yellow perch aged 2–5. Specifically, Lp_50_s increased over time (i.e., fish were predicted to become mature at larger sizes for a given age) across all ages (Figures 5 and 6). On average, Lp_50_ increased by ~100 mm from the beginning of the time series to the end. For example, in the early 1980s, age‐4 yellow perch had a 50% probability of maturing around 150 mm TL, while by the mid‐2010s this size had increased to 220–250 mm TL. Patterns in these increasing trends appeared to vary among ages (Figure 6). For age‐2 and age‐3 fish, Lp_50_ estimates increased initially, were relatively stable immediately following the fishing closure, and showed some evidence for an increase in the 2010s. For both ages, Lp_50_ posterior overlaps between the mid‐1980s and mid‐1990s was < 10%, while the overlap between the mid‐1990s and mid‐2010s cohort Lp_50_ was 30%–35% (Figure S13). In age‐4 and age‐5 fish, there was less change from the 1980s to the late 1990s or early 2000s (Lp_50_ posterior overlaps 35%–45%) and a clearer increase after the fishery closed (Lp_50_ posterior overlaps between the late‐1990s or early‐2000s and mid‐2010s were 10%–20%, Figure S13). This increase was also apparent in our ability to reliably model PMRNs for older age classes. In early years, very few age‐4 or age‐5 fish were immature, leading to few reliable PMRN estimates, whereas by the mid‐2000s a more even mixture of immature and mature individuals in these age classes allowed PMRNs to be estimated for more cohorts (Figures 6 and S12).

## 4 Discussion

The process of maturation in fishes represents a dynamic synthesis of multiple plastic and evolutionary responses to biotic and abiotic effects on growth, reproductive investment, and mortality (Roff 1984; Cottingham et al. 2018; Waters et al. 2021). Here, we used a Bayesian hierarchical approach to PMRN estimation to account for plasticity in maturation caused by the effects of changing temperatures, system productivity, and commercial harvest on Lake Michigan yellow perch, allowing for a higher‐resolution time series of maturation shifts. After accounting for plastic responses to these variables, maturation was found to occur at larger sizes (e.g., PMRN midpoints were larger) across cohorts. This increase was more pronounced among older age fish (age‐4 and ‐5) after a commercial fishing moratorium was put in place, supporting the idea that earlier fisheries‐induced evolution toward maturation at smaller sizes and younger ages had previously occurred in this population. These results are a relatively rare demonstration of how the relaxation of formerly intense harvest can spur an evolutionary response toward recovery of pre‐harvest life history traits (Conover, Munch, and Arnott 2009; Swain and Mohn 2012), especially in freshwater fish where few examinations have occurred (Edeline et al. 2009).

We detected multiple strong plastic effects of individual phenotype on size at maturity, all of which were operating according to previous observations and supported by life history theory, namely positive effects of total length and age, which interacted to reduce the importance of length at older ages (Stearns and Koella 1986). The effect of length was considerably larger than any other predictor in the model, which may align with previous suggestions that yellow perch maturation is largely length‐based, and therefore changes in growth rates could allow for significant plasticity in age at maturity (Feiner et al. 2017). The interaction between age and length also aligns with theory—as fish age, the cost of delaying maturation, and therefore risking mortality before having a chance to reproduce, increases, meaning attaining large size should become less important at older ages (Stearns and Koella 1986; Nilsson‐Örtman and Rowe 2021). Interestingly, we did not detect a strong effect of body condition (relative weight) on maturity, despite previous research suggesting that increased body condition should result in maturation at younger ages and smaller sizes (Grift et al. 2007; Mollet, Kraak, and Rijnsdorp 2007; Uusi‐Heikkilä et al. 2011; Feiner, Shaw, and Sass 2019). This may suggest that, on average, body condition was generally sufficient for maturation in this population, or that the effects of changing resource availability were more strongly evinced through changes in growth.

Of the ecosystem‐level predictors considered, only the presence or absence of the commercial fishery considerably influenced maturity in this population. The probability of fish being mature was higher in years when the commercial fishery was active (until 1996) than when the commercial fishery was closed (1997 onward). High fishing pressure should reduce population sizes and intraspecific competition, fueling faster growth and more precocious maturation schedules through density‐dependent plasticity. When fishing is reduced, increased population sizes may slow growth and delay maturity. Multiple species have exhibited such a response to changes in fishing pressure, including percids (Gobin et al. 2016; Haglund, Isermann, and Sass 2016; Denechaud et al. 2020). It is also possible that contemporaneous changes in resource availability would have the same effect; the invasion of *Dreissena* in particular significantly reduced primary productivity around the same time (Nalepa, Fanslow, and Lang 2009). However, we attempted to account for variation in resource availability using measurements of total phosphorus and found little evidence of an effect on maturity status. This may suggest that the effects of resource availability on maturation were largely explained by the effects of age, length, and condition (which incorporated the effects of resource availability via its effects on growth or energetic reserves). That a large effect of fishing remained in the maturity model, and that PMRN midpoints continued to exhibit pre‐ and post‐fishing differences, suggests that fisheries harvest was indeed a consequential driver of yellow perch maturation schedules during this time period.

Even after accounting for substantially more potential sources of plastic variation in maturation schedules and improving our method to estimate PMRNs on near‐annual time scales, we observed substantial increases in PMRN midpoints over time across all ages of female yellow perch. The largest sizes at maturation occurred near the end of the time series, approximately 3–4 generations following the closure of the commercial fishery, similar to changes observed by Feiner et al. (2015b). We suggest that this result strengthens the hypothesis that changes in PMRNs represent a possible evolutionary response to the cessation of the yellow perch commercial fishery in Lake Michigan. Changes in overall mortality rates, even in the absence of size selection, can result in corresponding changes in maturation (Swain and Mohn 2012; Olin et al. 2017), meaning reductions in mortality alone in this case could have led to the increasing size and age at maturation observed. Adding to this, reductions of intense size‐selective harvest could have allowed for increased fitness among large‐maturing genotypes as a result of positive maternal effects previously observed in yellow perch. Positive correlations between female size and offspring size and survival could contribute to positive selection for fish that delayed maturation and attained larger body sizes later in life (Collingsworth and Marschall 2011; Andree et al. 2015; Feiner et al. 2016). While we still cannot discount the possibility that such relatively rapid evolutionary responses were due in part to immigration or population replacement from other areas of the lake (as in Feiner et al. 2015a, 2015b), previous and more recent genetic and mark‐recapture studies have suggested a panmictic southern Lake Michigan population with little genetic input from other areas (Glover et al. 2008; Chorak et al. 2019). These results support previous assertions that Great Lakes yellow perch stocks are capable of rapid life history evolution.

Trends in Lake Michigan yellow perch maturation have been held up as a potential example of rapid evolutionary recovery of larger sizes at maturation following the closure of the commercial fishery and restriction of the recreational fishery in the late 1990s (Feiner et al. 2015b). Great Lakes yellow perch in general may exhibit the capacity to rapidly increase size at maturation when fisheries harvest is reduced, as this phenomenon was also observed in Lake Erie (Gíslason et al. 2018). However, limitations in previous studies mean these rapid changes need to be interpreted with caution. Feiner et al. (2015a, 2015b, 2017) estimated PMRN midpoints on a decadal timescale owing to relatively small annual sample sizes. In the last decade of the time series, reflecting the collapse of the population, annual sample sizes were sometimes in the tens of fish, contrasting studies in marine populations which routinely have access to observations of thousands or tens of thousands of individuals per cohort (Barot et al. 2004b). In addition, previous studies generally failed to account for dynamic ecosystem changes occurring in Lake Michigan around the same time as the fishery closure. For instance, multiple species invasions reduced system productivity and significantly altered the Lake Michigan food web (Bunnell et al. 2014; Madenjian et al. 2015), which could have influenced the growth and condition of yellow perch and therefore biased previous PMRN estimates.

The original design of PMRNs was to account for plastic variation in these life history traits to allow for the detection of the potential evolution of maturation, particularly in exploited fish populations. However, multiple studies have highlighted issues with PMRN estimation, particularly in their robustness with sparse data (Gobin, Fox, and Dunlop 2021) or use on populations experiencing myriad environmental stressors (Gíslason et al. 2019). Using a Bayesian hierarchical model framework, we were able to provide the clearest picture yet of life history dynamics in a valued sport fishery. Most importantly, our model allowed us to “share strength” among parameter estimates across years and cohorts with varying data availability. The hierarchical approach permitted the estimation of parameters for cohorts with limited data (e.g., post‐2000). We were also able to better account for the potential effects of water temperatures (Dabrowski et al. 1996; Ciereszko et al. 1997; Wright, Millar, and Gibb 2011), system productivity, commercial fishing, and body condition (Grift et al. 2007; Uusi‐Heikkilä et al. 2011) on the maturation process. In doing so, our model provides a framework for improved Bayesian PMRN estimation that could allow for more detailed investigations into the relative plastic and evolutionary processes driving life history patterns in exploited populations, particularly in inland waters which rarely have sufficient data for classic PMRN estimation and therefore are lacking in evaluations of life history evolution in response to fishing or other stresses (Dunlop, Feiner, and Höök 2018).

## 5 Conclusions

In this study, we developed an improved, hierarchical model structure to estimate PMRN midpoints while accounting for multiple biotic and abiotic covariates hypothesized to have influenced maturation schedules of yellow perch in Lake Michigan. By doing so, we confirmed previous research that suggests yellow perch have undergone rapid, evolutionary recovery in maturation schedules since the closure of the commercial fishery in the late 1990s. However, this potential trait recovery has not been associated with a recovery in recruitment or abundance—yellow perch remain at some of the lowest abundances in recorded history (Makauskas and Clapp 2018; Stacy‐Duffy et al. 2020). Females that mature at larger sizes and ages are often more productive, and a broader diversity of mature age‐ and size‐classes is generally thought to promote population productivity (Hsieh et al. 2010; Hixon, Johnson, and Sogard 2013; Feiner, Shaw, and Sass 2019). Our results, therefore, suggest that other barriers to yellow perch recovery in Lake Michigan remain. Important stressors, like climate change, losses in primary productivity, and changes in zooplankton communities, could be limiting larval or juvenile yellow perch survival (Stacy‐Duffy et al. 2020; Stadig et al. 2020; Zorn et al. 2020). Our results suggest, however, that should these barriers to larval survival be mitigated, Lake Michigan yellow perch may retain a high production capacity.

Our model structure opens the possibility for the application of PMRNs to detect life history evolution in a wider range of fish populations and fisheries, particularly in inland fisheries that suffer from relatively poorer data availability than marine species. For example, substantial efforts have been made to build regional and national databases of inland fishery survey data (e.g., Lynch et al. 2021; Frater et al. 2024), which could include sufficient information to model maturation processes over longer time periods or at multiple spatial scales by leveraging a hierarchical framework similar to what we have presented here. Future expansions of our hierarchical framework to include important temporal correlations in traits among cohorts, and better model the importance of covariance among multiple abiotic and biotic effects on maturation could improve the estimation of the pace of evolution in harvested populations. As we have shown, fisheries‐induced evolution is likely not limited to the intense marine fisheries where it is most often investigated. Other studies have shown the capacity for inland recreational fisheries to impose strong selection gradients, on, for example, fish aggression or brooding behavior (Cooke et al. 2007; Philipp et al. 2009). Pairing these observations with continuing work to evaluate correlations between PMRNs and changes in allele frequencies to understand the genetic underpinnings of maturation (Uusi‐Heikkilä et al. 2015, 2017), as well as expanding our understanding of the mechanisms driving intrapopulation variation in PMRNs, could lead to new avenues for integrating PMRNs into fisheries management and policy.

## Conflicts of Interest

The authors declare no conflicts of interest.

## Supporting information


Figures S1‐S13.

## Data Availability

Yellow perch data for this study are available at https://purr.purdue.edu/publications/1709/2. Abiotic data and code are stored in a publicly available GitHub repository at https://github.com/zsfeiner/Perch‐FIE.
