# Supplementary material for: Hierarchical Multi‐Dimensional Maturation Modeling to Isolate the Effects of Commercial Closure on a Great Lakes Fishery
Source: Evol Appl. 2025 Mar 4;18(3):e70075. doi: 10.1111/eva.70075 (PMC11880125; doi:10.1111/eva.70075)
Supplement: Supplementary file 1 — Figures S1‐S13. [file EVA-18-e70075-s001.docx]

**Supplemental Material**

**Hierarchical multi-dimensional maturation modeling to isolate the effects of commercial closure on a Great Lakes fishery. Evolutionary Applications.**


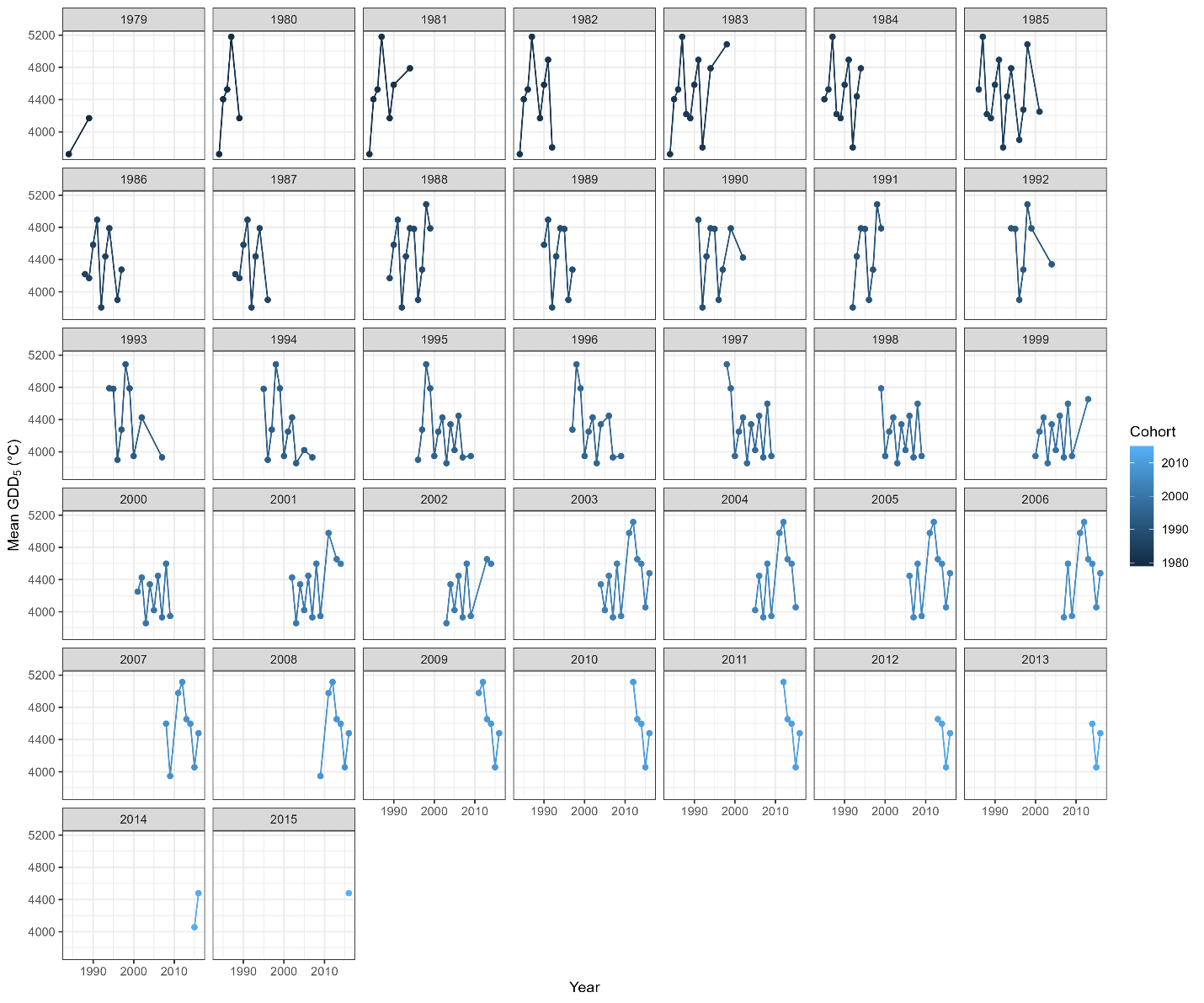


**Figure S1.** Mean annual growing degree days (base 5°C, GDD_5_) experienced by all cohorts included in maturation modeling in each year they were captured, demonstrating that cohorts experienced a range of interannual conditions interannually.


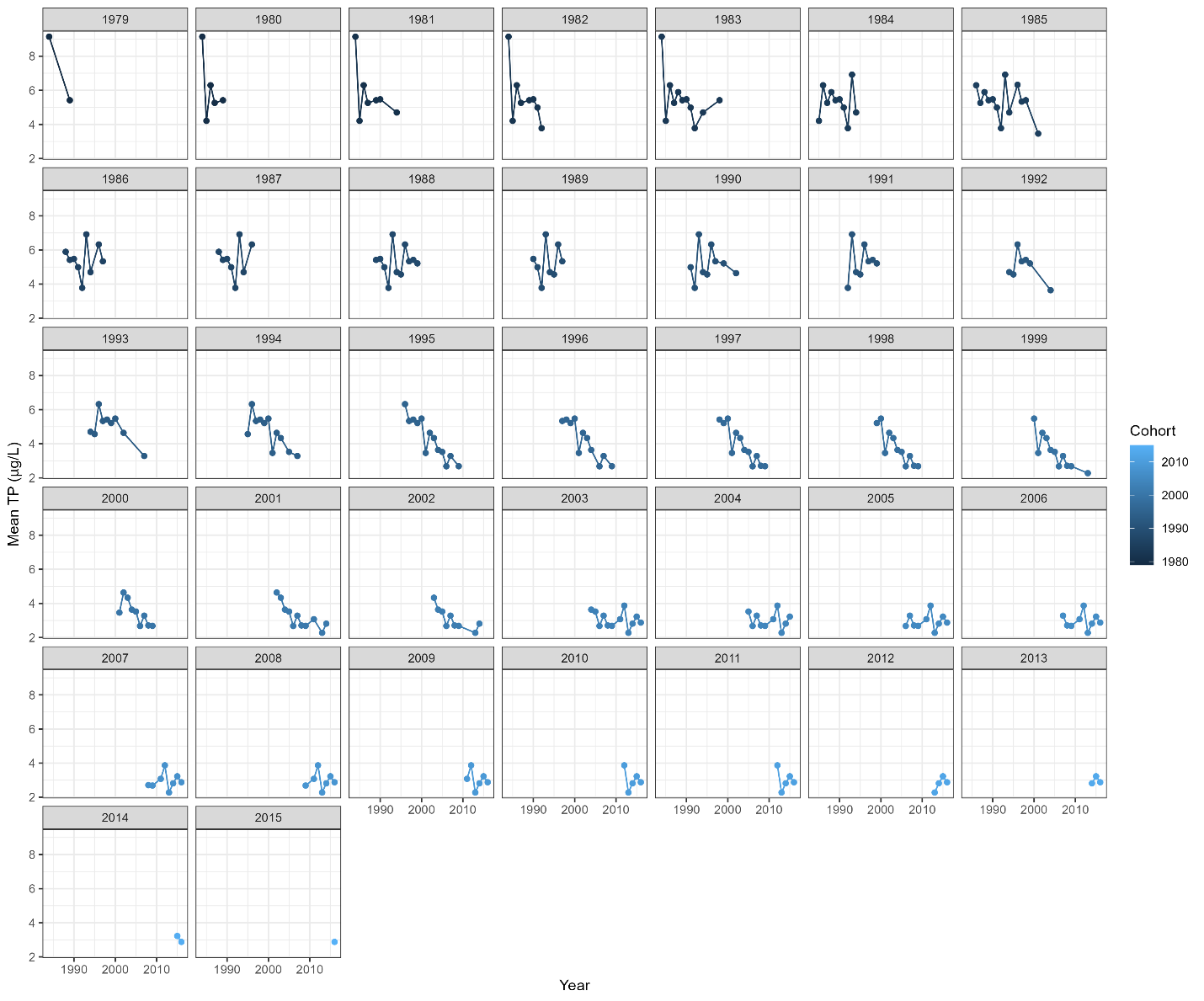


**Figure S2.** Mean total phosphorus (TP, μg/L) experienced by all Lake Michigan yellow perch cohorts included in maturation modeling in each year they were captured, demonstrating that cohorts experienced a range of interannual primary productivity.


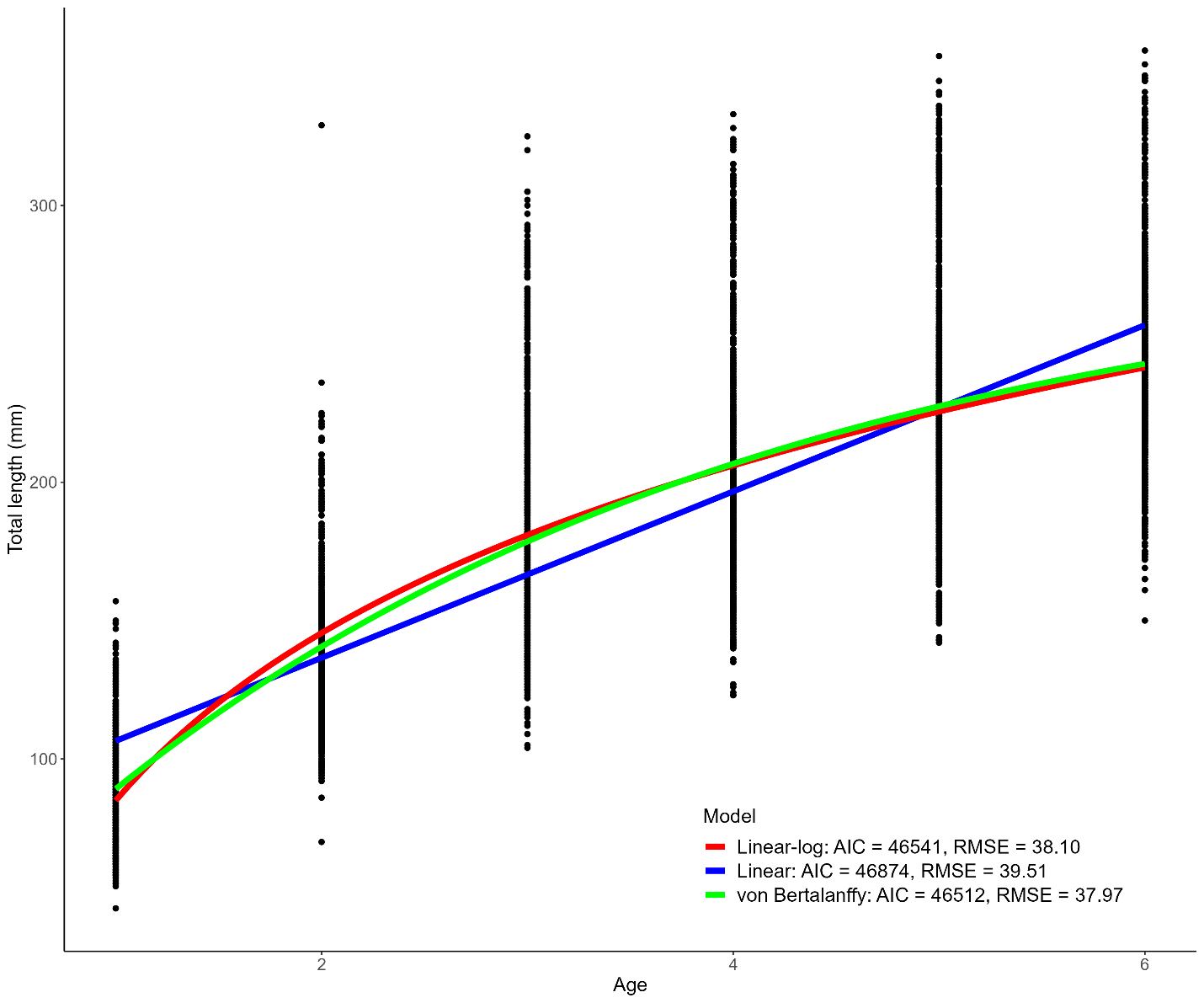


**Figure S3.** Comparison of models for female Lake Michigan yellow perch growth, limited to fish younger than 7 years of age (i.e., before complete maturity), including linear (blue), linear-log (red), and von Bertalanffy (green). Model AIC and root mean squared error (RMSE) are shown in the legend. The linear-log model was selected for use in probabilistic maturation reaction norm modeling (see main text) owing to its highly similar fit compared to the von Bertalanffy model and relative simplicity of implementation.


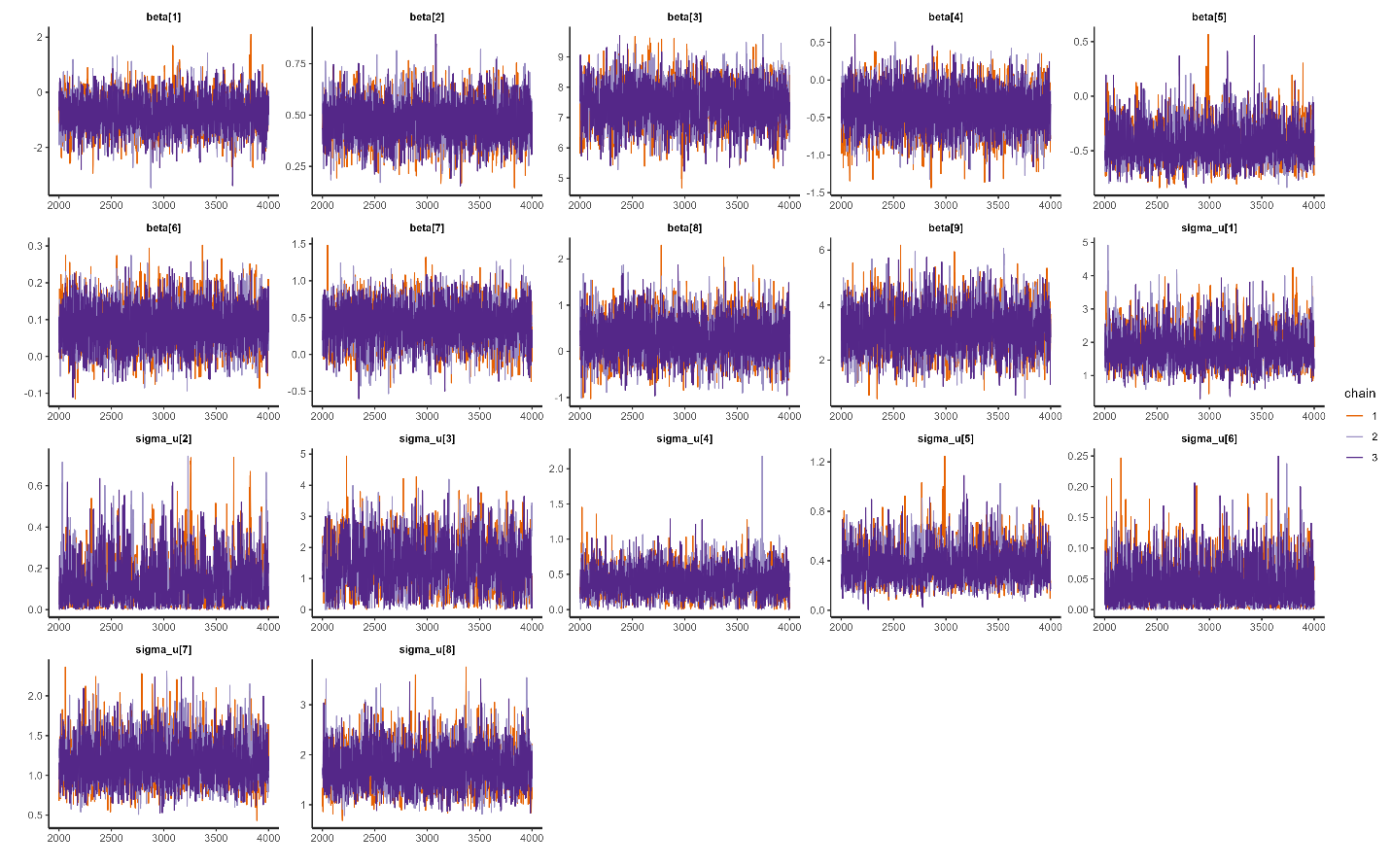


**Figure S4**. Traceplots for all model coefficients used to model Lake Michigan yellow perch maturity. beta[1:9] and sigma_u[1:9] are the coefficients and variances of for: (1) model intercept; (2) age (years); (3) total length (mm); (4) relative weight; (5) the interaction between total length and age; (6) the interaction between relative weight and age; (7) growing degree days; (8) total phosphorus; and (9) commercial fishing. See Table 1 in the main text for parameter definitions and priors.


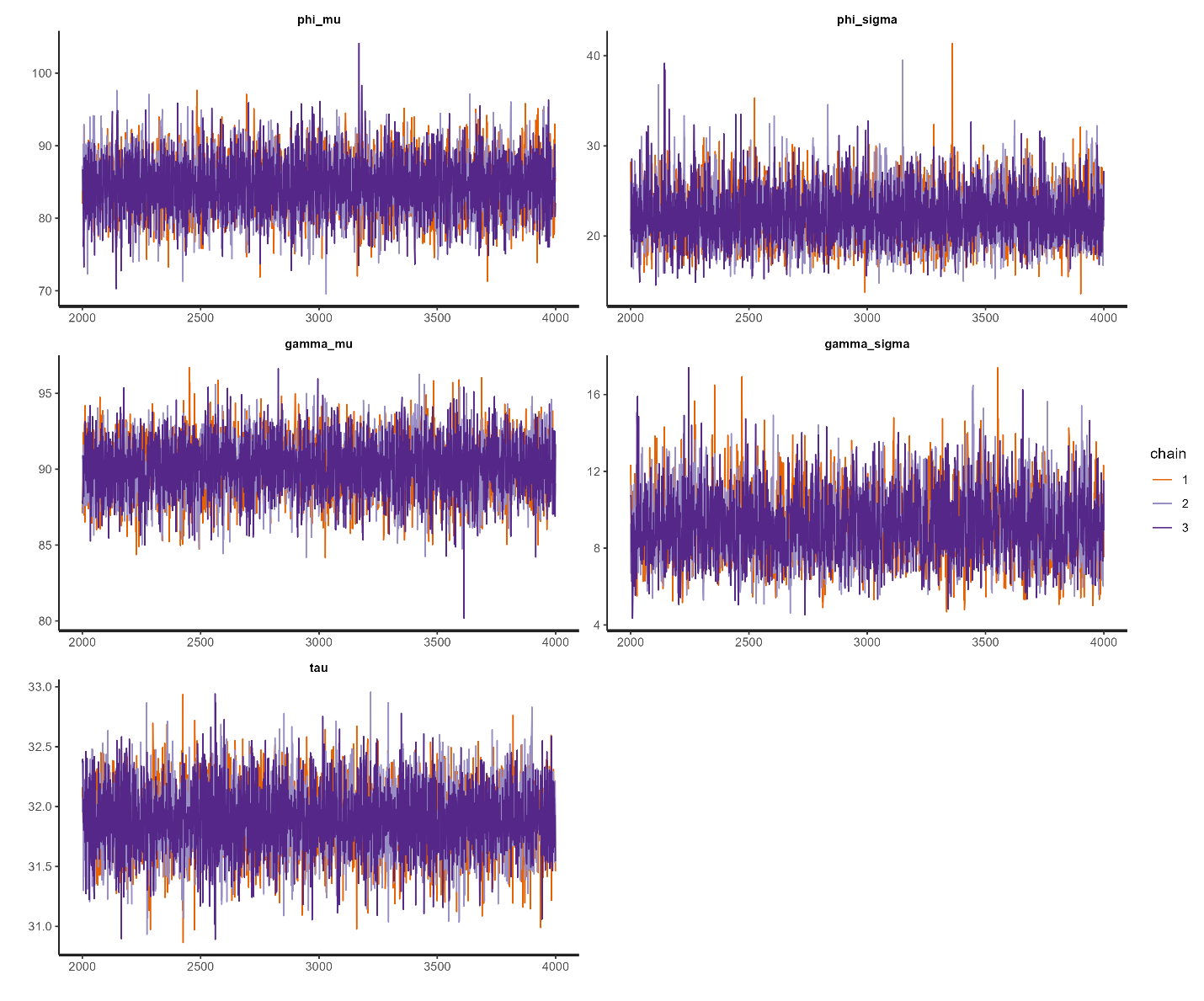
**Figure S5**. Traceplots for hyperpriors used to model Lake Michigan yellow perch growth, including the mean and standard deviation of the intercepts (phi_mu and phi_sigma), mean and standard deviation of age effects (gamma_mu and gamma_sigma), and model error (tau). See Table 1 in the main text for parameter definitions and priors.


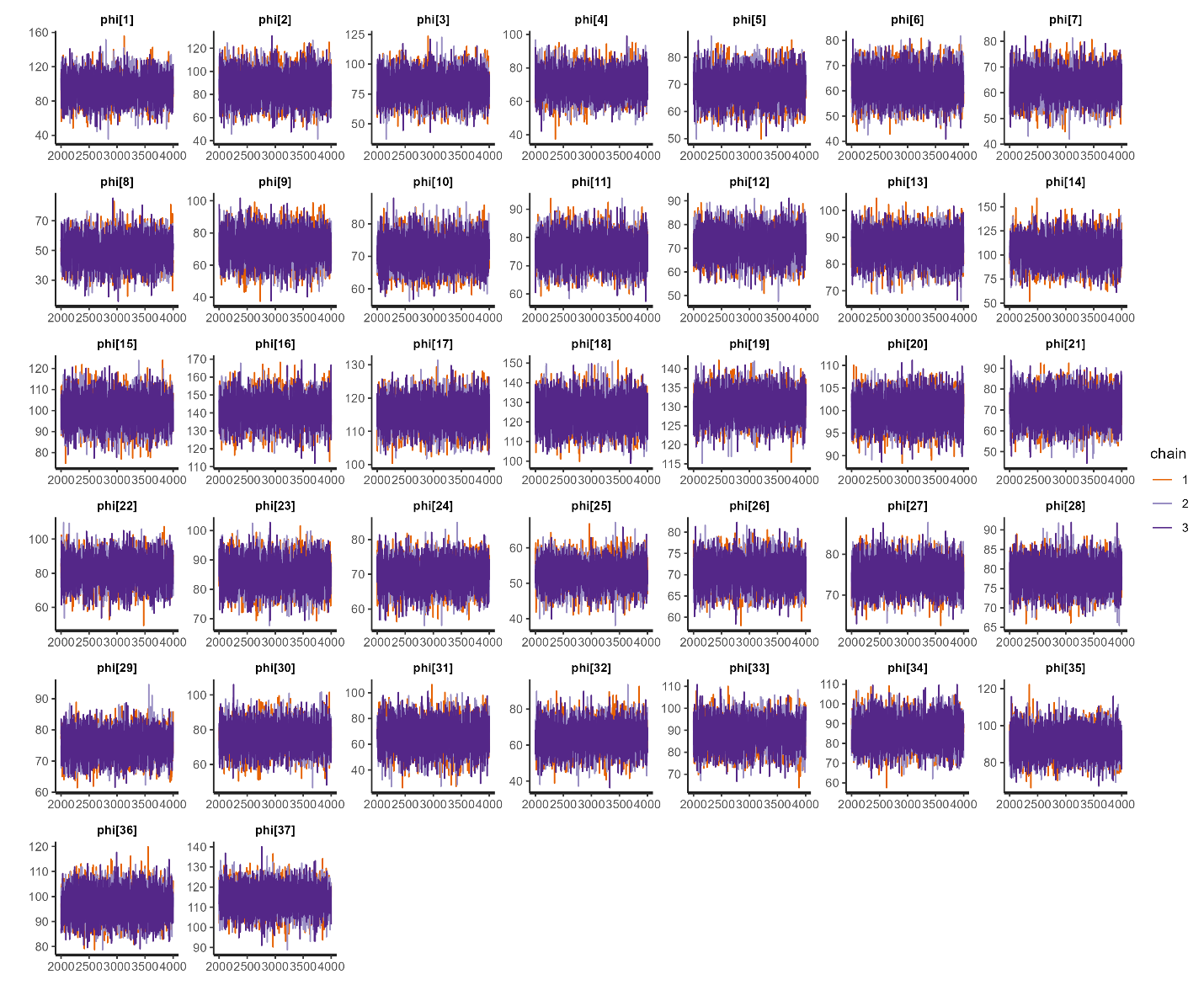


**Figure S6**. Traceplots for cohort-specific intercepts of growth in linear-log model of Lake Michigan yellow perch growth (phi[1:37]). See Table 1 in the main text for parameter definitions and priors.


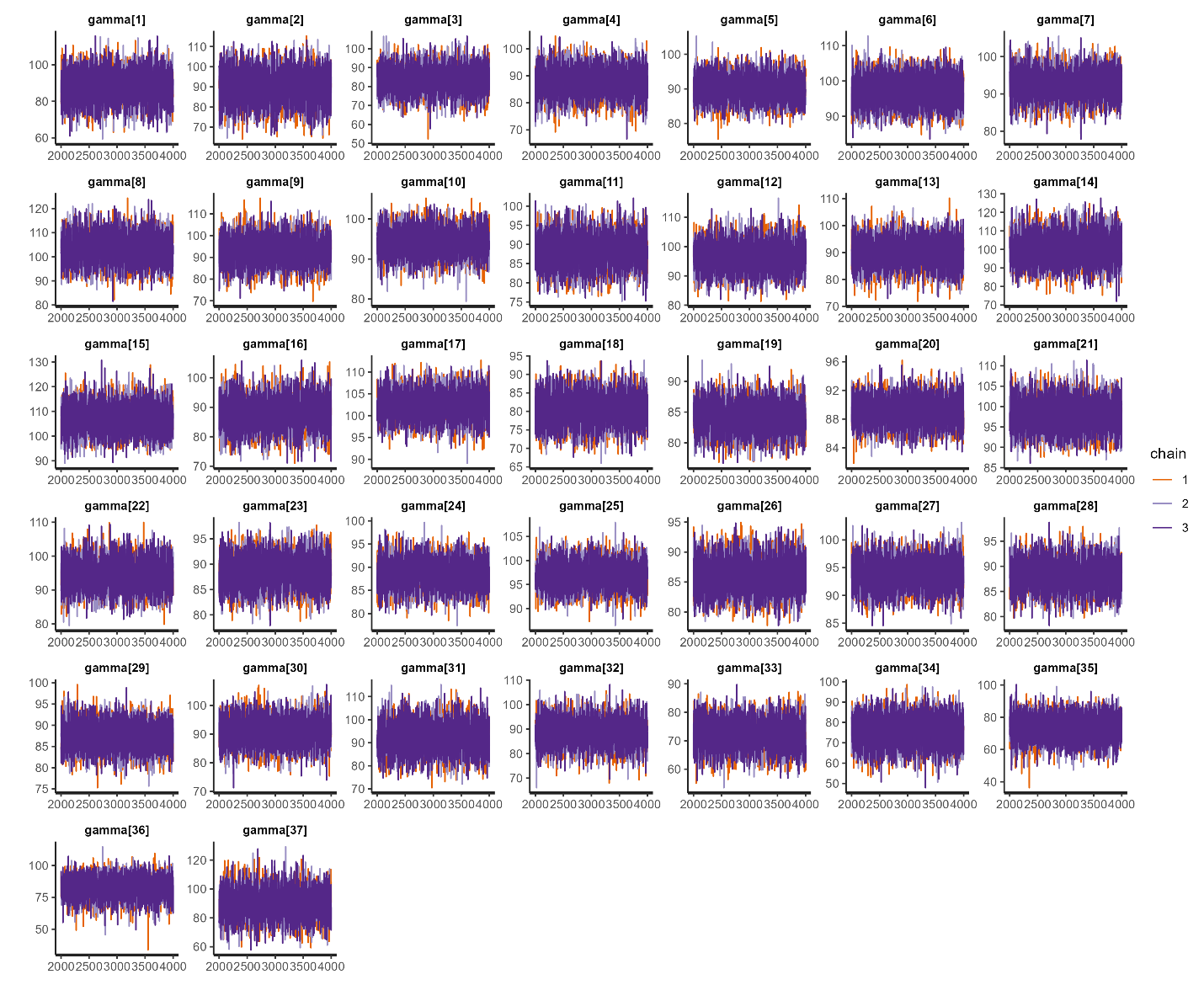
**Figure S7**. Traceplots for cohort-specific age effects on growth in linear-log model of Lake Michigan yellow perch growth (gamma[1:37]). See Table 1 in the main text for parameter definitions and priors.


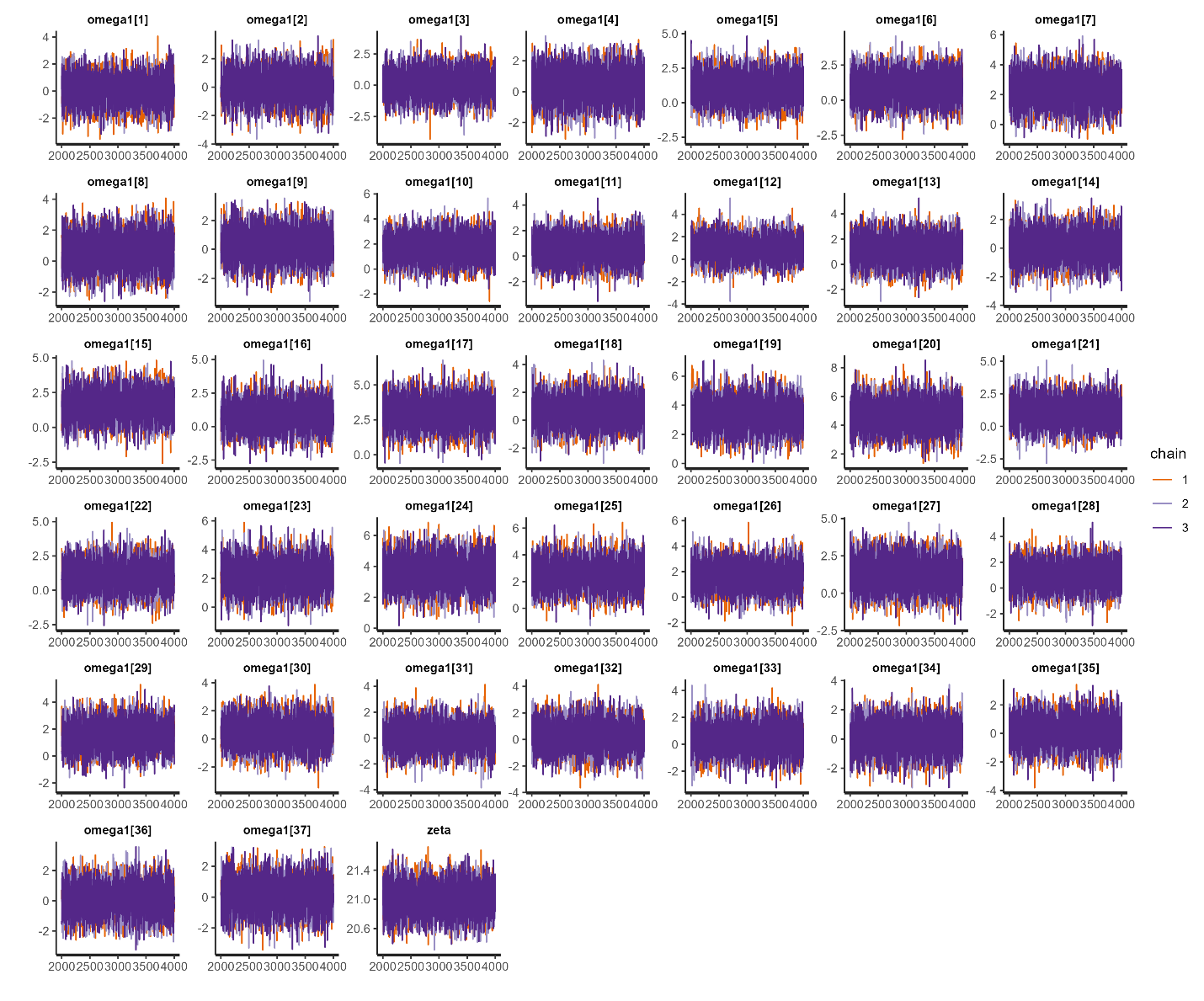
**Figure S8**. Traceplots for cohort-specific intercepts of model for body condition (omega1[1:37]) and condition model error (zeta). See Table 1 in the main text for parameter definitions and priors.


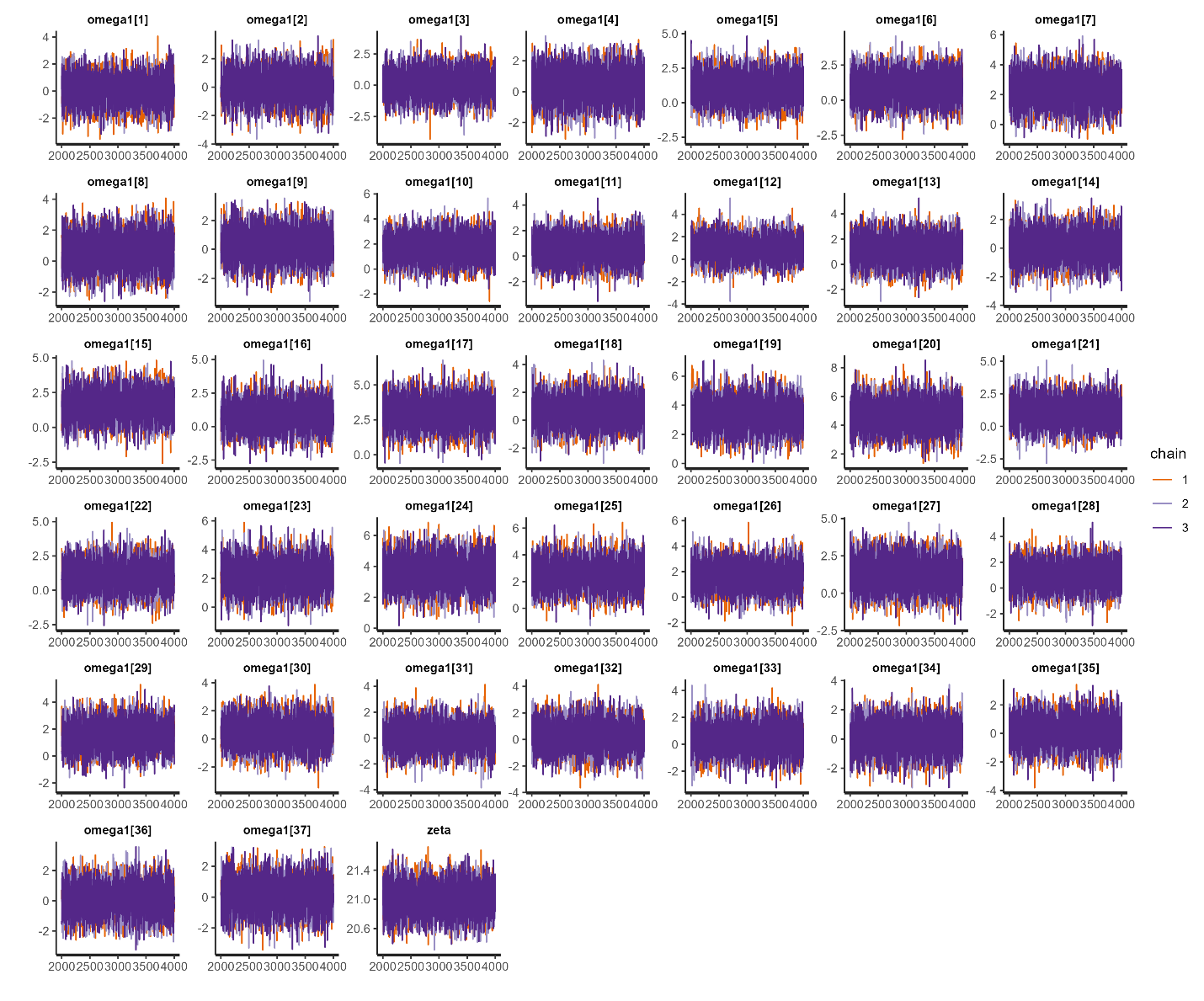


**Figure S9**. Traceplots for cohort-specific effects of age in model for body condition of Lake Michigan yellow perch (omega2[1:37]). See Table 1 in the main text for parameter definitions and priors.


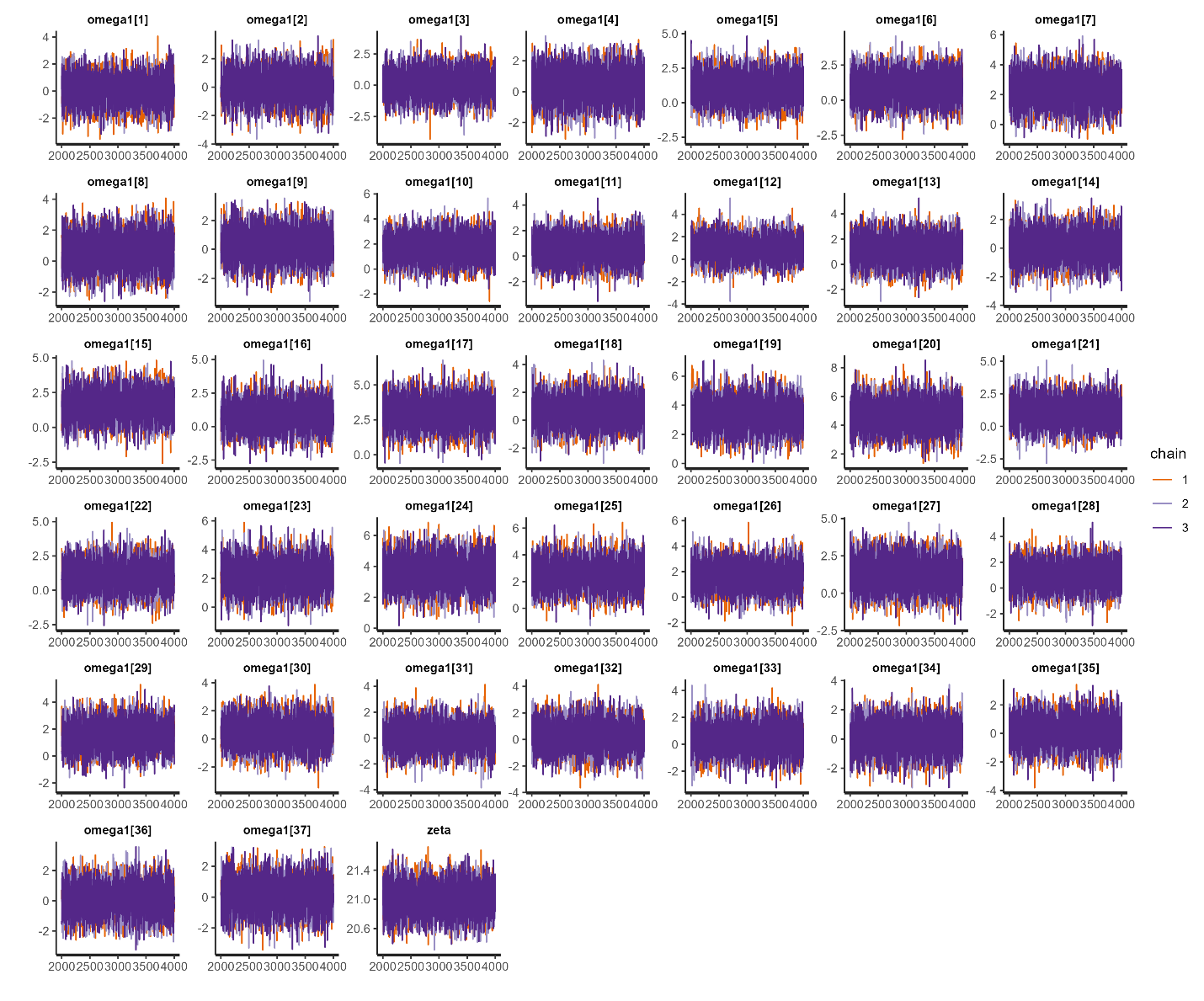


**Figure S10**. Traceplots for cohort-specific effects of length in model for body condition of Lake Michigan yellow perch (omega3[1:37]). See Table 1 in the main text for parameter definitions and priors.


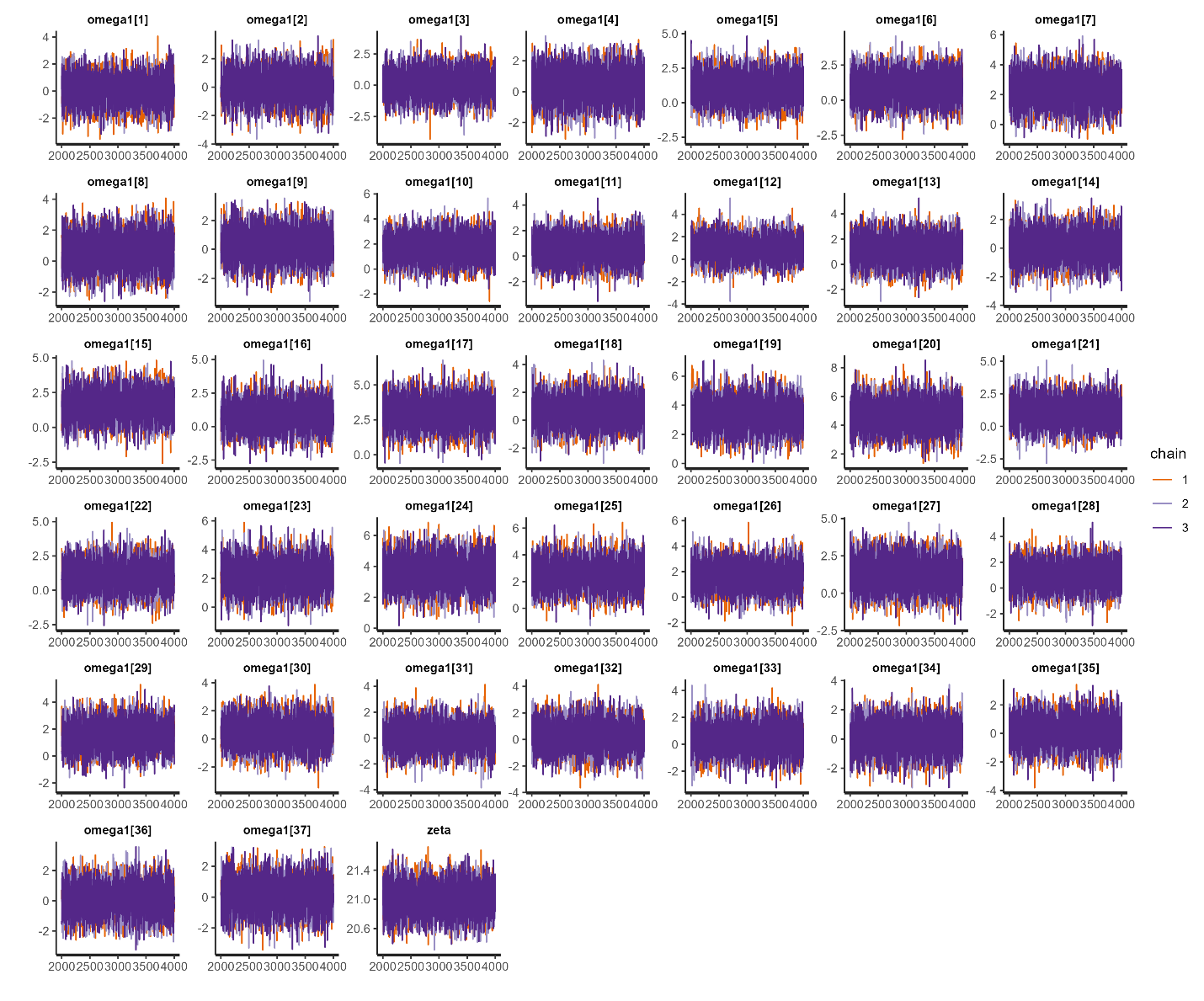


**Figure S11**. Traceplots for cohort-specific effects of the age*length interaction in model for body condition of Lake Michigan yellow perch (omega4[1:37]). See Table 1 in the main text for parameter definitions and priors.


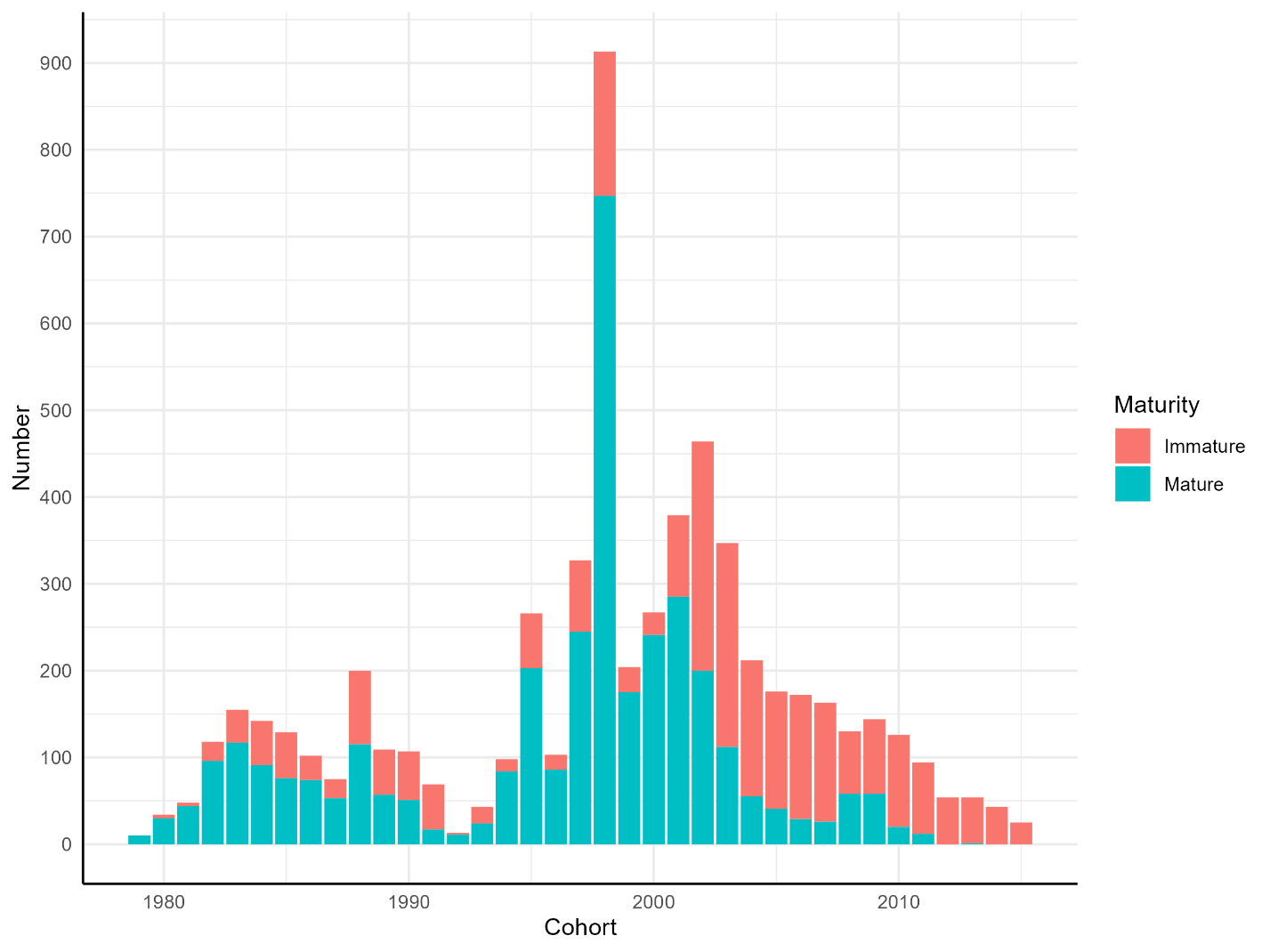
**Figure S12**. Sample sizes and numbers of female Lake Michigan yellow perch fish that were immature (red) or mature (blue) by cohort.


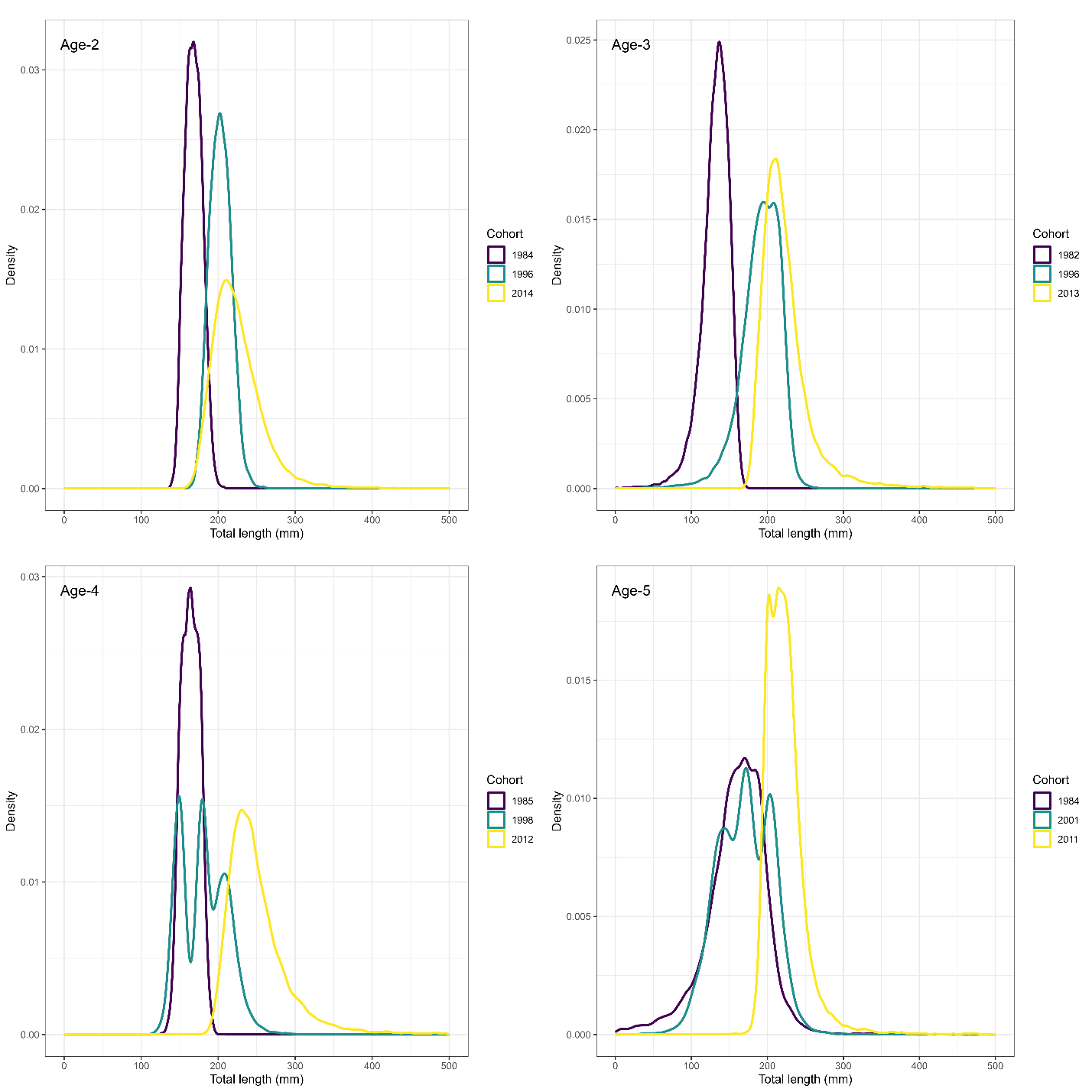


**Figure S13.** Full posterior distributions from 6000 posterior draws of a Bayesian hierarchical modelling of Lake Michigan yellow perch probabilistic maturation reaction norm midpoints (Lp_50_ in mm total length) for fish ages 2-5 (panels). Colors represent Lp50 posteriors from cohorts near the start of the timeseries (1982-1985), around the time commercial fishing in Lake Michigan was closed (1996-2001), and near the end of the timeseries (2011-2014). Low overlap of density distributions suggests that Lp_50_ changed over time.
